# Modulatory mechanisms of TARP γ8-selective AMPA receptor therapeutics

**DOI:** 10.1038/s41467-023-37259-5

**Published:** 2023-03-25

**Authors:** Danyang Zhang, Remigijus Lape, Saher A. Shaikh, Bianka K. Kohegyi, Jake F. Watson, Ondrej Cais, Terunaga Nakagawa, Ingo H. Greger

**Affiliations:** 1 Neurobiology Division, MRC Laboratory of Molecular Biology, Cambridge, UK; 2 Department of Molecular Physiology and Biophysics, Vanderbilt University, School of Medicine, Nashville, USA; 3 Present Address: IST Austria, Klosterneuburg, Austria

**Keywords:** Ion channels in the nervous system, Molecular neuroscience, Ligand-gated ion channels, Permeation and transport

## Abstract

AMPA glutamate receptors (AMPARs) mediate excitatory neurotransmission throughout the brain. Their signalling is uniquely diversified by brain region-specific auxiliary subunits, providing an opportunity for the development of selective therapeutics. AMPARs associated with TARP γ8 are enriched in the hippocampus, and are targets of emerging anti-epileptic drugs. To understand their therapeutic activity, we determined cryo-EM structures of the GluA1/2-γ8 receptor associated with three potent, chemically diverse ligands. We find that despite sharing a lipid-exposed and water-accessible binding pocket, drug action is differentially affected by binding-site mutants. Together with patch-clamp recordings and MD simulations we also demonstrate that ligand-triggered reorganisation of the AMPAR-TARP interface contributes to modulation. Unexpectedly, one ligand (JNJ-61432059) acts bifunctionally, negatively affecting GluA1 but exerting positive modulatory action on GluA2-containing AMPARs, in a TARP stoichiometry-dependent manner. These results further illuminate the action of TARPs, demonstrate the sensitive balance between positive and negative modulatory action, and provide a mechanistic platform for development of both positive and negative selective AMPAR modulators.

## Abstract (web summary)

AMPA receptors associated with TARP subunits enable the development of selective AMPA receptor drugs. Here, the authors provide cryo-EM structures of receptors bound to three TARP-γ8 selective drugs, and reveal bifunctionality of one ligand.

## Introduction

AMPARs mediate the majority excitatory synaptic transmission in the brain, and are central to the synaptic plasticity that underlies learning^1^. Their dysfunction (promoting both hypo- and hyper-excitability) is associated with multiple neurological and neuropsychiatric disorders, including memory deficits, epilepsy, depression, pain, motoneuron disease and gliomas^1–3^. Due to their molecular diversity, AMPARs generate a rich repertoire of post-synaptic response properties^1,4^, consequently offering sites for selective therapeutic intervention^5–9^. Four core subunits (GluA1-4) arrange into receptor tetramers as two non-equivalent pairs (termed AC and BD) that fulfil different functions^10,11^. Further diversity comes from an array of auxiliary subunits, associating with the receptor in various stoichiometries, and in a circuitry-dependent fashion^4,12–15^.

Major auxiliary subunits encircle the ion channel periphery, including the TARPs (transmembrane AMPAR regulatory proteins), CNIHs (cornichon homologs), and GSG1L (germline-specific gene 1)^1,16,17^. TARPs are the most prevalent, and constitute an integral component of most AMPARs. Resembling tetraspan claudins^18^, TARPs have four transmembrane helices and an extracellular, five-stranded beta-sheet harbouring flexible loops^10,19,20^. Based on protein sequence and function, three groups exist, Type 1a (TARPs γ2, γ3), Type 1b (γ4, γ8), and Type 2 (γ5, γ7), which are expressed in overlapping yet distinct brain regions, differentially impacting gating kinetics, ion conductance, pharmacology, and trafficking^4,12,13,16,21^.

**Fig. 1:**
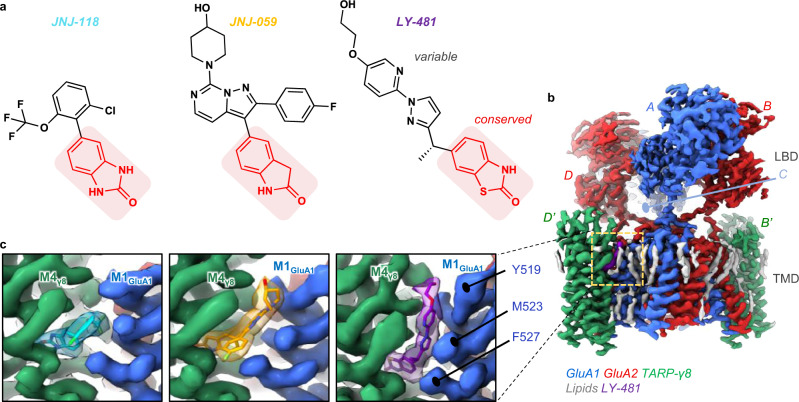
Cryo-EM maps of three NAMs bound to the resting-state GluA1/2_γ8 AMPAR. **a** Chemical structures of the three NAMs. The shared oxindole isostere is labelled red, the variable groups are shown in black. **b** Cryo-EM map of the GluA1/2_γ8 LBD (Ligand Binding Domain) and TMD (Transmembrane Domain) sectors, associated with LY-481. The colour code of protein, lipid and NAM is indicated below the map. Subunits are also labelled as described in the text (GluA1: A, C; GluA2: B, D; TARP γ8: B’, D’). **c** Cryo-EM maps of each NAM in side view. TARP γ8 (helix M4) is shown in green, GluA1 (helix M1) in blue. Also shown are three GluA1 residues involved in NAM co-ordination (Tyr519, Met523, Phe527).

A maximum of four TARPs associate with the receptor through two pairs of binding sites (termed A’C’ and B’D’) (Fig. 1), formed by the gate-surrounding M1 and M4 helices^19,20^. The more accessible B’D’ sites are preferentially occupied by the bulkier TARPs (Type 1b and Type 2)^22,23^, and by GSG1L^24^, while Type 1a TARPs and CNIHs have no obvious site preference^19,25^. TARP stoichiometry determines receptor function, as the twofold symmetry of the receptor dictates that the two pairs of binding sites provide different access for the TARP to the gating machinery^19,22^, consisting of the M3 gate, the gating linkers, and the ligand-binding domains (LBDs). TARP interactions with the receptor are complex: the TARP M3 and M4 helices engage the ion channel through transmembrane contacts, while their extracellular loops mediate transient contacts with the LBD and the gating linkers^1,16,26^. How this arrangement generates the wide spectrum of TARP modulation remains to be resolved.

As major excitatory receptors, AMPARs are central drug targets. However, the majority of available drugs target the GluA core subunits, thus causing severe side effects due to their ubiquitous presence across the brain. These include negative allosteric modulators (NAMs) such as the anti-epileptic perampanel^27,28^, and positive allosteric modulators (PAMs) that are being developed as cognitive enhancers^29,30^. Auxiliary subunit diversity offers scope for the development of region-selective AMPAR therapeutics. Recently discovered NAMs target γ8, a TARP strongly enriched in the hippocampus, and show promise in pre-clinical epilepsy studies^31,32^. One example, LY-3130481 (CERC-611), is also effective in the treatment of pain and of gliomas^33,34^, with Phase-2 clinical trials currently ongoing (https://clinicaltrials.gov/ct2/show/NCT04714996). Understanding the binding modes and allosteric mechanisms of these drugs, will not only facilitate the refinement and development of therapeutics, but also enables unlocking the complexity of TARP modulation of the AMPAR.

Here, we present high-resolution cryo-EM structures of GluA1/2 TARP-γ8 AMPAR complex, associated with three diverse TARP-γ8 drugs, differing in size and potency - LY-3130481, JNJ-55511118, JNJ-61432059 (herein referred to as ‘LY-481’, ‘JNJ-118’, ‘JNJ-059’), occupying the same binding pocket. Differential action of the ligands are based on the reorganization of molecular interactions in the vicinity of the pocket, which propagate throughout the entire AMPAR-TARP interface. We also document an unexpected example for this NAM pocket mediating ligand-selective PAM action, depending on core subunit identity and TARP-γ8 stoichiometry. Collectively, these results advance our understanding of AMPAR modulation by TARP-γ8, and aid the development of specific AMPAR therapeutics.

## Results

LY-481 and JNJ-118 were identified in high-throughput screens, while JNJ-059, a more potent ligand, was developed subsequently (Fig. 1a)^31,32,35,36^. All three compounds have a shared oxindole group, which appears fundamental to TARP-γ8 binding in our previous study^37^. To understand and compare the binding modalities of these compounds, we obtained cryo-EM structures of each NAM with resting-state GluA1/2 receptors (Table S1), containing two γ8 subunits at the preferred B’D’ sites (by tethering γ8 to GluA2; Methods) (Fig. 1b). JNJ-059 was also captured in an active state, with an open channel gate. Resolutions of the ion channel/TARP sector at **∼**3.0 Å, with the LY-481 structure reaching 2.6 Å (Supplementary Movie 1), enabled an unprecedented view of the modulator binding site with associated lipids and putative water molecules (Supplementary Figs. 1–4).

In addition, we subjected these structures to large scale MD simulations in an explicit lipid membrane together with water and ions, alongside previously reported apo-state GluA1/2_ γ8 complexes (open and resting; PDB: 7QHB and 7OCD, respectively)^22,38^. Three sets of simulations for each receptor, totaling 1.25–1.5 μs of sampling for each system, offered additional insight into ligand behavior in their binding pocket, as well as enabling a comprehensive comparison of local and global receptor dynamics in response to NAM binding (as shown for LY-481 in Supplementary Movie 2).

### Ligand binding mode

The NAM binding pocket locates near the boundary of the lipid bilayer with the extracellular milieu (Fig. 1b, c). MD simulations reveal water molecules penetrating into the pocket from γ8 Ser128 on the extracellular edge down to γ8 Asn172 in the binding pocket (Supplementary Fig. 4a), suggesting a potential route for ligand entry, with access via the lipid bilayer offering an additional route^,^, as also suggested in a recent study of JNJ-118^39^. Putative waters appear in the LY-481 cryoEM map around GluA2 Ser790, and in the NAM pocket at γ8 Ser128 (Supplementary Fig. 4b and c), which overlaps with water seen in the simulation (Supplementary Fig. 4a–c).

**Fig. 2:**
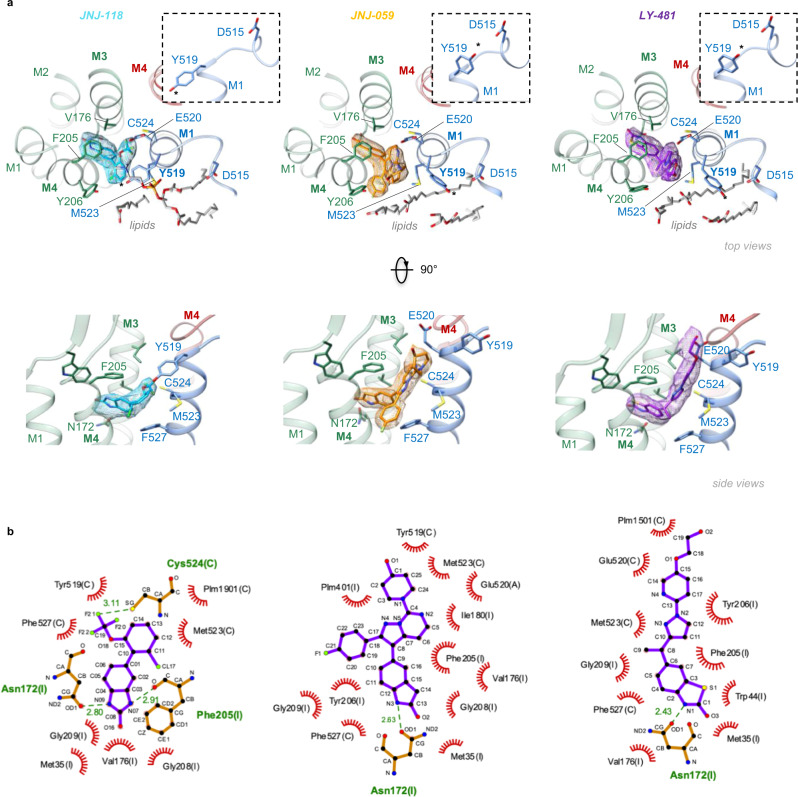
NAM binding pockets and coordinating residues. **a** Top panels: Top view onto the NAM pockets. TARP γ8 is shown in green, AMPAR helices are blue (GluA1 M1) and red (GluA2 M4), the three NAMs are colour-coded. Ligand-interacting residues and lipids are shown in stick. The insets show side views onto Tyr519, which swings towards Asp515 in the JNJ-059 and LY-481 structures (Tyr519 are also highlighted with asterisks). Bottom panel: as above but rotated 90˚. **b** Ligplot diagrams of the three NAMs^73^. H-bonds are indicated as green, dashed lines, while non-bonded contacts with the ligands are denoted by red spoked arcs.

Modulator selectivity for TARP-γ8 is determined by two residues unique to γ8, Val176 and Gly209, in the M3 and M4 helices^31,32^. These positions are occupied by bulkier residues in the other TARPs (Ile and Ala, respectively), restricting ligand access (Supplementary Fig. 4d). All three NAMs anchor between these two γ8 residues through a common oxindole isostere secured by a H-bond between γ8 Asn172 and an amine of the oxindole (Fig. 2 and Supplementary Fig. 4e), consistent with our recent docking study^37^, and replicating a previously reported binding mode of JNJ-118^40^. The oxindole’s benzene ring is capped by γ8 Phe205, further stabilizing the ligand. MD simulations show that the ligands remain stably bound in this site throughout 500 ns runs (Supplementary Fig. 4f).

**Fig. 3:**
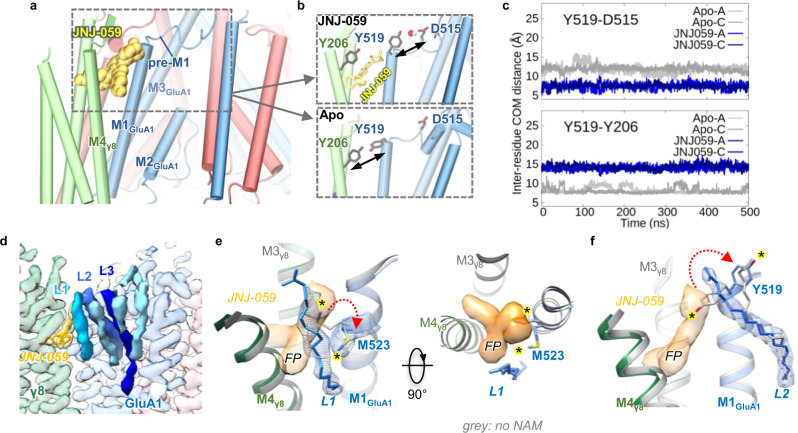
JNJ-059-induced H-bond formation, and lipid interactions. **a** Model of the receptor complex with JNJ-059 (yellow spheres), highlighting the proximity between the NAM pocket and the GluA1 pre-M1 helix (the figure is derived from an MD simulation). **b** JNJ-059 disrupts Tyr519 interaction with Tyr206 (γ8) (top panel), as compared to a simulation without the NAM (‘Apo’; bottom panel), but enables an H-bond between Tyr519 and Asp515 in the pre-M1 helix (top). **c** Variations in distance between centres of mass for Tyr519-Asp515 and Tyr519-Tyr206 in MD simulations of resting-state models. Distances are compared for the apo state (grey) and the JNJ059-bound structure (blue). Data are for both GluA1 subunits (chains A and C) shown in light and dark colours. **d** Cryo-EM map of the resting-state JNJ-059 structure. Lipid densities stacking along the GluA1 pre-M1 helix are shown in shades of blue (L1-3). **e**, **f** Overlay between the JNJ-059-resting model (coloured) with an apo resting state (PDB: 7OCD; grey). **e** The NAM induces an interaction between Met523 and the acyl chain of lipid L1 (side view, left; top view right), and a rearrangement of L2 via the Tyr519 side chain (**f**). NAM-induced side chain reorientations are highlighted with an asterisk. The flurophenyl group interacting with L1 is denoted ‘FP’. Note the dilation of the NAM pocket in the presence of JNJ-059.

While oxindole groups are shared, and their binding modes analogous, the remainder of each compound is variable. These variable moieties project towards the top of the GluA1 M1 helix, increasing the distance between M1 and the TARP-γ8 M4 helix, and triggering side chain reorientations of Tyr519, Met523 and Phe527 (Fig. 2a). The same residues rearrange to engage γ8 upon its binding to the receptor^10^. Together with Glu520 and Cys524, they form the main ligand coordination points on the AMPAR (Fig. 2b). In the JNJ-118 structure, the top of the binding site is capped by Tyr519, which points towards γ8 Tyr206, comparable to ligand-free structures. By contrast, the two larger NAMs reach towards Glu520 at the lipid-solvent interface, and force the Tyr519 side chain towards Asp515 in the pre-M1 helix (Fig. 2a). Pre-M1 surrounds the M3 gating helices^11^, and dilates together with the M3 gate on channel opening^22,38^, thus forming a key regulatory element immediately adjacent to the NAM pocket (Fig. 3a). Tyr519 persistently engages Asp515 through a water-mediated H-bond in MD runs with JNJ-059 or LY-481 bound, but this interaction is not observed with either a ligand-free structure (PDB: 7OCD), or with the smaller JNJ-118 ligand, where Tyr519 engages γ8 Tyr206 instead (Fig. 3a–c). Notably, a putative H-bond between Tyr519 and Asp515 is also apparent in our high-resolution LY-481 structure (Supplementary Fig. 5a). We hypothesize that this bond impacts the local dynamics between pre-M1 and the M3 gate, perhaps by influencing a hydrophobic interaction between the neighboring Pro516 (in pre-M1) and Phe619 (in M3). These two highly conserved residues are implicated in gating regulation in NMDA receptors^41,42^, and in coordinating the negative allosteric AMPAR modulators GYKI and perampanel, which bind the core subunits directly^43,44^.

### Impact of JNJ-059 on opening of the gate

How these NAMs ultimately affect opening of the gate is currently elusive. To address this, we determined an open-state structure of GluA1/2_γ8 associated with JNJ-059, which was overall similar to an open-state without the modulator (PDB: 7QHB); with a root mean square deviation of 0.36 Å, when aligning Cα atoms of the AMPAR/TARP transmembrane sector (Supplementary Fig. 5b). When focussing onto the gating core, opening of the M3 gate was still apparent in the presence of JNJ-059, although the gate constriction point (at GluA1 Thr621/GluA2 Thr625) was slightly wider without the NAM (by ~0.5 Å) (Supplementary Fig. 5c and d). Therefore, even the bulkiest NAM does not compromise an open gate conformation, underscoring mechanistic differences to NAMs targeting the core receptor (such as perampanel)^44,45^. Based on a resting state structure in complex with JNJ-118, it has been hypothesized that NAM binding precludes outward movement of the M3 gating helices;^40^ however, the highly similar architecture of active states in the absence and presence of a NAM suggests other contributors underlying NAM action, as investigated below.

### Lipids shape the NAM-binding pocket

The binding pocket opens sideways into the lipid bilayer, enabling ligand interactions with lipids, which stack nearby along the horizontal pre-M1 helices (Fig. 3d)^10^. The fluorophenyl group of JNJ-059 projects towards the acyl chain of a lipid (L1) (Fig. 3e), which is also engaged by the distal hydroxy-ethyloxy portion of LY-481, close to its (currently unresolved) head-group (Supplementary Fig. 5e). In the JNJ-118 structure, L1 arches above Tyr519, effectively capping the binding pocket (Supplementary Fig. 5f). L1 is also contacted by the displaced Met523 side chain in all three NAM structures, while the neighbouring lipid (L2) reorients around the dynamic Tyr519 side in response to LY-481 and JNJ-059 (Fig. 3e, f and Supplementary Fig. 5e). Hence, the three NAMs perturb the local lipid environment with the AMPAR in different ways. As these lipids (L1-L3) link the pre-M1 helix to the helices lining the conduction path: the M2 pore helix of the selectivity filter, and the M3 gating helix (Supplementary Fig. 5g)^22^, ligand-induced lipid reorganisation is a possible contributer to NAM action.

### Interfering with NAM action

**Fig. 4:**
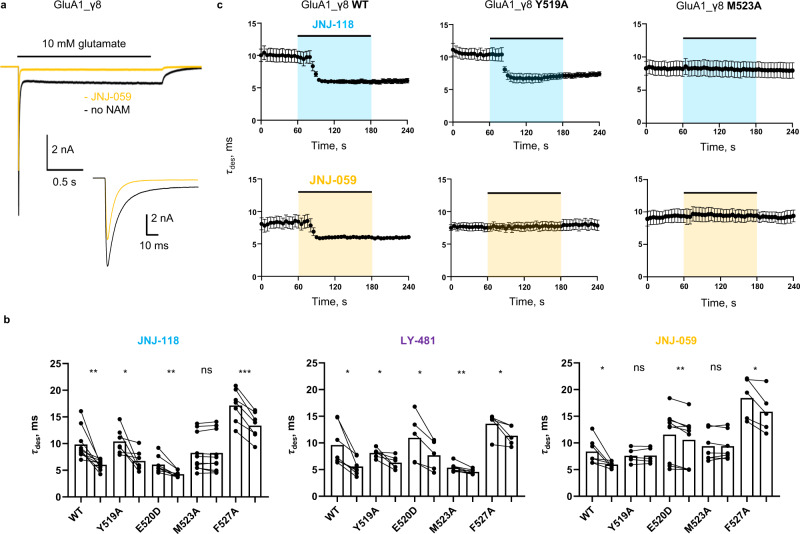
Functional NAM effects on wild type and mutant GluA1_γ8. **a** Whole-cell response of GluA1_γ8 to 10 mM glutamate (2 s; -60 mV), in either the absence (black) or presence (yellow) of JNJ-059. The inset zooms into the peak response. **b** Paired bar plots showing effect of 10 µM JNJ-118, LY-481 or JNJ-059 on desensitization time constant for wild type or mutated GluA1i_γ8. Each point is a measure of parameter in absence or presence of modulator. Bar height represents the mean value. Asterisks indicate summary of two-tailed paired *t*-test values: *p ≤ 0.05, **p ≤ 0.01, ***p ≤ 0.001 and ‘ns’ for *p* > 0.05. Number of cells: JNJ-118: *n* = 10, 7, 8, 10, and 7; LY-481: *n* = 7, 6, 5, 8, and 5; JNJ-059: *n* = 7, 5, 9, 7, and 5 for wt, Y519A, E520D, M523A, and F527A, respectively. **c** Time course of desensitization entry in response to JNJ-118 (top row) and JNJ-059 (bottom) row for GluA1_γ8 (left), Y519A mutant (middle) and M523 mutant (right). Scatter plot of average desensitization τ in time indicating the time-course of JNJ-059 or JNJ-118 effect on wild-type, M523A or Y519A GluA1i_ γ8. Black circles and whiskers indicate average values and standard error of the mean (SEM). Number of cells are *n* = 10 for GluA1i_γ8, *n* = 7 for GluA1i _γ8 Y519A, *n* = 10 for GluA1i_γ8 M523A with JNJ-118 and *n* = 7 for GluA1i_γ8, *n* = 5 for GluA1i_γ8 Y519A, *n* = 7 for GluA1i_γ8 M523A with JNJ-059. Source data are provided as a Source Data file.

To assess AMPAR modulation by the three NAMs comparatively we used patch-clamp recordings of recombinantly expressed receptor/TARP complexes. First, we generated a baseline for the action of γ8, using whole-cell recordings of GluA1 fused to γ8 (denoted GluA1_γ8). As expected, all three NAMs (at 10 μM) reduced the positive modulation conferred by γ8: reducing peak current amplitude, quickening desensitization kinetics, and decreasing equilibrium current and resensitization (Fig. 4a–c, and Supplementary Fig. 6a, b). Together, these changes reduce charge transfer through the channel. Despite their structural differences, at 10 μM the extent of NAM action was broadly similar for all three NAMs.

Differences became apparent when mutating side chains in the M1 helix that are displaced by the NAMs (Fig. 4b, c, and Supplementary Fig. 6b). At the top of M1, mutation of the conformationally variable Tyr519 to alanine (Y519A) appears to abolish negative modulation of desensitization kinetics by JNJ-059, but not JNJ-118 and LY-481 (Fig. 4b, c), suggesting that ligand-mediated projection of the Tyr519 side chain toward the pre-M1 helix could be critical for JNJ-059 action. Mutation of Met523 (M523A) essentially blunted NAM action of both JNJ-118 and JNJ-059 but not of LY-481 (Fig. 4b, c). At this position, all NAMs break the GluA1 Met523 interaction with γ8 Phe205, and force the Met523 side chain into lipid (Fig. 3e); the lipid interactions induced may contribute to negative modulation, and are lost by mutation to the shorter alanine side chain (M523A). By contrast, F527A and the E520D mutation, which ruptures an H-bond between Glu520 and γ8 Tyr201, retained NAM action on desensitization kinetics across the three ligands (Fig. 4b). The three NAMs exhibited a complex response pattern to these mutations for two other measures, the equilibrium current and resensitization (Supplementary Fig. 6b). Together, modulatory action is sensitive to features of the NAM variable groups, and their interaction pattern with the AMPAR M1 helix, implying ligand-specific local signalling routes.

### Endogenous oxindoles target the NAM pocket

The naturally occurring tryptophan metabolites isatin and 5-OH-indole are structurally remarkably similar to the γ8 docking group of the NAMs. These compounds also docked between the γ8 M3 and M4 helices (the cognate NAM site) in MD simulations, and remained bound throughout the run (Supplementary Fig. 7a). However, we observed no functional effect of isatin binding in patch-clamp recordings with GluA1_γ8 at concentrations up to 1 mM, and preincubation of isatin did not impact the efficacy of JNJ-118 (Supplementary Fig. 7b, c). Therefore, despite their structural similarity and ability to dock to the NAM pocket, lack of the variable group renders these compounds ineffective.

### Modulatory role of the γ8 transmembrane sector

**Fig. 5:**
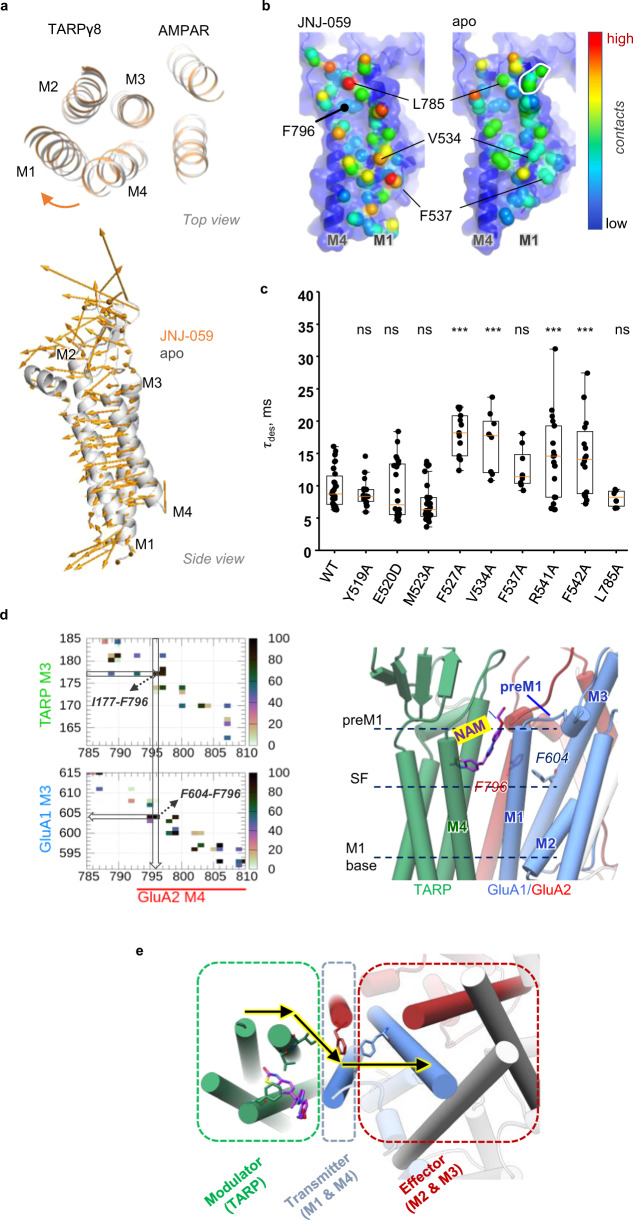
NAMs trigger a global reorientation of TARP γ8. **a** JNJ-059-induced rotation of γ8 helices M1, M2 and M4, relative to an apo structure (PDB: 7OCD). Alignment of the TMD sector of apo (grey) and JNJ-059-bound (orange) resting-state models. The vectors indicate the direction of γ8 in response to the NAM in both top and side views. Vectors were generated using the ‘modevectors’ script in PyMol. **b** TARP γ8 contact points along its binding site, the M4_GluA2_ and M1_GluA1_ helices. Contacted residues are coloured depending on the number of atoms contributing to the interaction (red: high; blue: low). Countacts were computed using ‘findNeighbors’ in ProDy’ with a 4.5 Å cutoff between heavy atoms^74^. **c** Box plots showing macroscopic desensitization τ for GluA1_γ8 wild type and mutants; each point is a τ_des_ (WT: 9.7 ± 0.6 ms, *n* = 30; Y519A: 8.9 ± 0.5 ms, *n* = 18; E520D: 9.4 ± 1.0 ms, *n* = 22; M523A: 7.4 ± 0.6 ms, *n* = 27; F527A: 18.0 ± 0.9 ms, *n* = 13; V534A: 17 ± 2 ms, *n* = 8; F537A: 13 ± 1 ms, *n* = 8; R541A: 16 ± 1 ms, *n* = 24; F542A: 15 ± 1 ms, *n* = 21; L785A: 8.0 ± 0.5 ms, *n* = 6; mean ± SEM) obtained by fitting the decaying phase of whole-cell currents with a single exponential. Boxes show the 25th/75th percentiles and whiskers indicate the furthest points that fall within 1.5 times of interquartile range from the 25th/75th percentiles. The horizontal line in each box shows the median value. Asterisks summarize one-way ANOVA test, Dunnett correction was used for multiple comparisons to wild type receptor (***p ≤ 0.001 and ‘ns’ for *p* > 0.05). **d** High-frequency residue contacts forming a potential pathway from the NAM binding site to the gate. Left panel: TARP-GluA2 (top) and GluA2-GluA1 (bottom) contact maps for the JNJ-059 resting state MD simulation, suggesting a route from TARP Ile177 via GluA2 Phe796 to GluA1 (see “Methods” for further detail). Middle panel: Pathway residues relative to key regions in the AMPAR pre-M1, selectivity filter (SF), and cytosolic base of M1. **e** Overall model suggesting that allosteric information from the TARP (Modulator) is communicated via the AMPAR peripheral M1 + M4 helices (Transmitter) to the M3 gate (Effector). Source data are provided as a Source Data file.

The structural changes upon NAM binding are not limited to *local* alterations of side chain orientations. We also observe *global* changes in the arrangement between the receptor and the TARP, which likely contribute to negative modulation. Specifically, a vertical outward rotation of γ8 helices in NAM-bound structures, relative to apo structures, is apparent (Fig. 5a). This motion was most prominent with JNJ-059, and includes the peripheral M1 and M2 helices as well as the top of M4, but not M3, which likely acts as a pivot axis. The clockwise rotation (when viewed from the top) could counter the motion of the TARP towards the receptor that accompanies channel activation^22^, and may thereby compromise transition to the active state, and/or its stabilization.

The three NAMs also alter the interaction landscape between γ8 and its binding site, the GluA1 M1 and GluA2 M4 helices. Contrary to what could be expected, contact analyses reveal *enhanced* γ8 interaction points with the receptor in the NAM structures, rather than a decoupling of γ8 (in comparison to apo structures PDB: 7OCD and 7OCE) (Methods) (Fig. 5b and Supplementary Fig. 7d). Strengthened contacts include Phe527, Val534 and Phe537 on the M1 helix, as well as Leu789, Val800 and Met807 on M4. Side chain interactions immediately adjacent to the binding pocket are either lost (Tyr519, Met523) or are unaltered (Cys524). This NAM-triggered realignment of the TARP likely alters the overall energetics of the receptor complex.

To extend these findings, we examined AMPAR-TARP interaction dynamics through all-atom MD simulations. We computed AMPAR-TARP contacts, and generated difference maps between simulations from NAM-bound versus an apo structure (PDB: 7OCD) (Methods). This analysis further illuminated the NAM-induced rearrangement of the complex; while contacts around the NAM pocket are lost (GluA1 Tyr519, Glu520, Met523), the base of GluA1 M1, around Phe542, forms more persistent interactions with the γ8 M4 helix. Meanwhile, the γ8 M3 helix improves its overall engagement with GluA2 M4 at Leu789/Ser790, Val800, Met807 (Supplementary Fig. 7e). The enhanced contacts at the base of GluA1 M1 with the TARP extend into the cytoplasmic loop connecting M1 with the M2 pore helix, which forms the selectivity filter. Phe542, and the neighbouring Arg541, re-orient their side chains on γ8 binding^22^, with Arg541 poised to engage the negatively charged M1/M2 loop. These two M1 residues have a substantial impact on γ8 modulation, as we show below.

To address whether the rearrangement of γ8 observed in Fig. 5b contributes to modulation, we mutated multiple GluA1 residues, scattered throughout the binding-site, and recorded their functional effects in the absence of NAMs. This analysis again revealed a complex picture with different effects on γ8 function: M523A and E520D tended to reduce positive modulation by the TARP, by contrast, other mutants further slowed desensitization kinetics and increased the equilibrium response (Fig. 5c and Supplementary Fig. 8a). Positive TARP modulation is facilitated by F527A and by mutants in the lower part of the M1 helix, including V534A, F537A, R541A and F542A. Overall, this pattern of changes in desensitization correlates with M1-TARP interaction strength—the upper part of M1 is loosely coupled to γ8, while the region below Phe527 is coupled more tightly^38^. Moreover, effects on resensitization were distinct to the ones observed for desensitization, where R541A in particular blunted the resensitization component (Supplementary Fig. 8a), highlighting that different regions of the receptor contribute to this measure.

Lastly, we find that the enhanced TARP modulation is not immune to NAM action, as it is also blunted by all three NAMs when mutating positions distal from the binding pocket, as shown for R541A and F542A (Supplementary Fig. 8b). Therefore, in addition to the more intensively studied TARP loops, our analysis highlight transmembrane interactions in TARP modulation, which are sensitive to perturbation of specific contact points.

### Mechanism of NAM action

Taken together, we propose that γ8 NAMs signal through a combination of local and global routes. Due to their proximity to the gate, local perturbations can be transmitted effectively, via the top of M1 and the pre-M1 helix (Fig. 3a). These perturbations include side chains of binding pocket residues, such as Met523 and Tyr519, as well as associated lipids stacking along pre-M1 (Figs. 2a and 3d–f). This gate-proximal region is engaged differently by other major AMPAR auxiliary subunits, such as CNIHs and GSG1L. By influencing either transition to the active state, or the stability of the open state conformation^22^, they generate their unique modulatory profiles^22,25,46,47^. NAMs target this strategic location and interfere with this process.

In addition, the NAM-triggered realignment of the TARP can be communicated across the entire γ8 binding-site (the M1, M4 helices) and thereby to core elements of the conduction path, the M3 gate and the M2 pore loop. Possible transmission pathways between contact points emerge from MD simulations, where residues contributing to a given pathway are identified through stable interaction times during the MD simulations (i.e. high percentage of residue pair interactions within 4 Å, excluding hydrogens) (Fig. 5d**;** left panel). One observed route leads from the ligand co-ordinating Val176/Ile177 in γ8, via Phe796 (in the GluA2 M4 helix) to Phe604 in the M3 helix (Fig. 5d). Phe796 is strategically located within van der Waals contact distance of both the TARP and the M3 helix (at Phe604), as well as the NAM coordinating Cys524 (Figs. 2c and 5b). Towards the cytoplasmic end of the M1 helix, residues sensitive to modulation by γ8 (such as Phe537; Fig. 5c) directly contact the M2 pore loop of the selectivity filter and will thereby influence the lower half of the conduction path (Fig. 5d**;** right panel), while Arg541 targets the acidic M1/2 cytoplasmic loop, implicated in AMPAR regulation^38,48^. In summary, contacts in the AMPAR-TARP transmembrane sector appear to be finely tuned for transmission via the M1/M4 periphery (‘transmitter’) to the ‘effector’ (the M3 gate and M2 selectivity filter) (Fig. 5e). This interaction landscape is perturbed by NAM binding.

### JNJ-059 acts as a PAM on GluA2

**Fig. 6:**
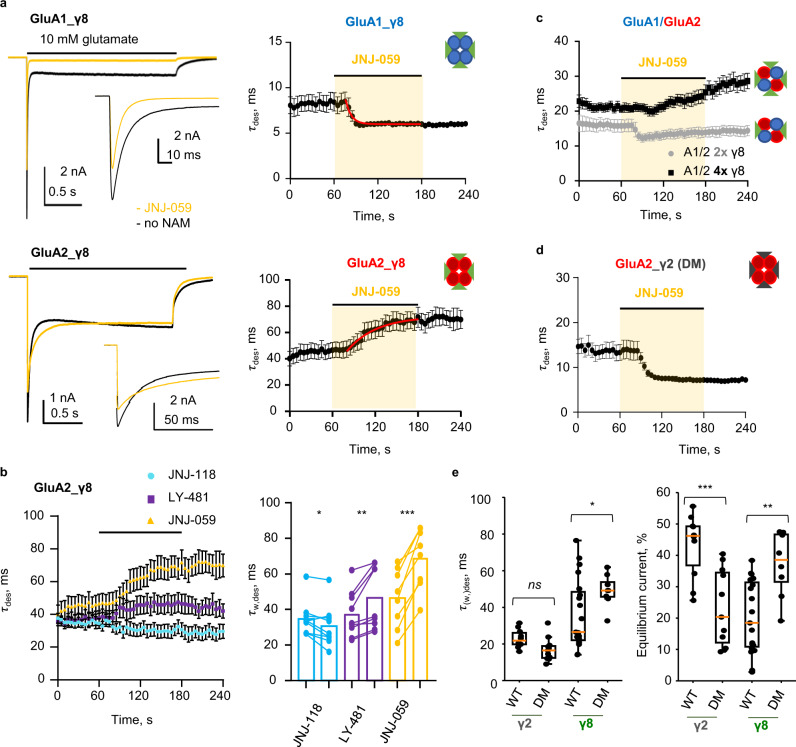
JNJ-059 PAM action on GluA2-containing AMPARs associated with four γ8 subunits. **a** Left: Representative whole-cell responses to 10 mM glutamate (2 seconds, −60 mV) from HEK293T cells transfected with GluA1_γ8 (top; repeated from Fig. 4 to facilitate direct comparison) or GluA2Q_γ8 (bottom) tandem in control condition (black) and in presence of JNJ-059 10 µM (orange). Insets show initial phase of currents at faster time scale. Right: Scatter plot of average desensitization τ over time indicating the time-course of JNJ-059 effect on GluA1_γ8 (*n* = 7; top; repeated from Fig. 4c) or GluA2Q_γ8 (*n* = 8; bottom). Black circles and whiskers indicate average values and SEM. Red line is a fit of the pooled data with a single exponential (τ = 7.2 ms and 40.4 ms for GluA1_γ8 and GluA2Q_γ8, respectively) for display purposes only. **b** Left: Scatter plot of average desensitization τ in time indicating the time-course of modulator effect on GluA2Q_γ8. Black circles and whiskers indicate average values and SEM. Number of cells are *n* = 9 for JNJ-118, *n* = 9 for LY-481 and *n* = 8 for JNJ-059. Right: Summary paired plots showing the effect of all three modulators on the τ_w,des_ for GluA2Q_γ8. Bars represent mean values. Number of cells as on left. Asterisks indicate summary of two-tailed paired t-test values (*p < = 0.05, **p < = 0.01, ***p < = 0.001). **c** As in **a**, for GluA1/GluA2R_γ8 (*n* = 4; grey circles) and GluA1/GluA2R_γ8/ γ8 (*n* = 10; black squares). **d** As in **a**, for GluA2Q_γ2DM (*n* = 5). **e** Box plots showing macroscopic desensitization τ (left) and equilibrium current (right) for GluA2Q_γ2 or γ8 (wild-type or double mutant (DM; I153V/A184G in γ2, V176I/G209A in γ8)). Each point for the τ_(w,)des_ plots was obtained by fitting the decaying phase of whole-cell currents with one (γ2) or two (γ8, weighted τ_des_ is presented) exponentials. *n* = 10, 11, 19, and 8 for γ2, γ2DM, γ8, and γ8DM, respectively. Boxplots as described in Fig. 5C. Asterisks indicate summary of two-tailed not-paired t-test values (*p < = 0.05, **p < = 0.01, ***p < = 0.001 and ‘ns’ for *p* > 0.05). Source data are provided as a Source Data file.

We obtained an unexpected result when testing JNJ-059 on a GluA2_γ8 homomer (GluA2 fused to γ8), which was opposite to what was seen with the GluA1_γ8 homomer. Contrary to the speeding of desensitization, which is expected by interference with γ8’s modulatory action, JNJ-059 triggered a slowly developing PAM response with GluA2_γ8, manifested in further slowing desensitization kinetics (Fig. 6a). JNJ-059 also failed to reduce the GluA2_γ8 equilibrium current, but still abolished resensitization (Supplementary Fig. 9a). This behaviour was not seen with JNJ-118, which acted as a NAM on GluA2 for all measures (Fig. 6b and Supplementary Fig. 9a, b), consistent with a recent study^39^. Similar to JNJ-118, LY-481 triggered a pronounced reduction of the equilibrium response, but modestly slowed GluA2_γ8 desensitization kinetics (Fig. 6b and Supplementary Fig. 9b). Hence, the two larger ligands failed to exert a full NAM effect on GluA2, with JNJ-059 binding at this site also generating PAM activity.

The slower kinetics of PAM development (~5-fold slower than NAM action; Fig. 6a, right panels) suggest that a different mechanism underlies its expression. In support of this, mutating the tyrosine on top of the M1 site in GluA2 (Y523A, the equivalent of GluA1 Tyr519) did not block PAM action (Supplementary Fig. 9c), despite its strong prevention of NAM action in GluA1. The same held for GluA2 Met527. While the equivalent mutation in GluA1 (M523A) robustly abolished NAM activity (Fig. 4b and c), GluA2 M527A did not blunt the GluA2 PAM phenotype (Supplementary Fig. 9c), suggesting that other receptor elements are recruited to elicit positive modulation by JNJ-059.

Despite the presence of GluA2, PAM activity was not apparent in the GluA1/2_γ8 heteromer associated with *two* γ8 subunits. This receptor responded to JNJ-059 with a NAM phenotype, comparable to GluA1_γ8 (Fig. 6c; grey data). Interestingly, PAM action was observed when co-expressing additional γ8 with GluA1/2_γ8 (Fig. 6c; black data). Therefore, contrary to NAM activity, population of all four TARP binding sites on the receptor is required for positive modulation of receptor kinetics. To determine if γ8 was an essential requirement, or whether any TARP could mediate positive modulation by JNJ-059, we created a NAM binding site on TARP-γ2, by mutating the non-conserved residues to those of γ8 (I153V, A184G) (Supplementary Fig. 4d)^31,32^. Surprisingly, GluA2 assembled with the γ2 double mutant (GluA2_γ2 DM) triggered a strong NAM response with JNJ-059 (Fig. 6d). Therefore, JNJ-059 PAM activity requires the presence of GluA2 and four TARP γ8 auxiliary subunits.

We note that the γ2 mutant, mimicking γ8 at the NAM binding site (I153V, A184G), was less potent in modulating GluA2 (Fig. 6e). On the contrary, mutating γ8 at these two positions, to match the other Type-1 TARPs (V176I/G209A), converted γ8 into a more efficacious TARP (Fig. 6e). This result further demonstrates the fine balance in modulatory activity, which can be achieved by specific binding modes at the TARP-AMPAR interface around the NAM binding site. This strategic region has been exploited in different ways by other auxiliary subunits to fine-tune the AMPAR response across various circuitries in the brain.

## Discussion

Our study offers two major insights. First, it provides an in-depth characterization and a mechanistic understanding of the modulatory action of TARP-γ8 drugs. Secondly, it illuminates the critical role played by the TARP transmembrane sector in modulating the AMPAR. Both insights will aid the development of therapeutics targeting these prevalent auxiliary subunit class.

Using three experimental approaches, we demonstrate that the action of these compounds is intricate and multi-modal, and is not simply due to an all-or-none occlusion of gate opening, as is the case for drugs directly targeting the AMPAR gating core^44,45^. This is in line with their modulatory (rather than inhibitory) activity, and highlights the unique niche for targeting the receptor periphery for more subtle and specific action. The diversity of AMPAR auxiliary subunits provides ample opportunity for modulator development in future screening campaigns^9^.

NAM binding to the AMPAR/TARP interface triggers both local (Figs. 2 and 3), and global effects (Fig. 5). The NAM pocket borders the pre-M1 helix and M4 gating linkers, both poised to effectively transmit any local perturbation directly to the M3 gate. This strategic location has been exploited by other auxiliary subunits. In CNIHs (CNIH1-3), three highly conserved phenylalanine side chains slot into a region overlapping the NAM pocket, and are central to positive modulation by CNIHs^22,25^. Interestingly, the negative modulatory GSG1L subunit harbors a bulky tryprophan side chain at this position, at a site analogous to γ8 Gly209 (the γ8-selective NAM anchor point). The functional impact of this residue is currently unknown. Although the precise allosteric routes leading from these elements to the M3 gate are not yet firmly delineated, multiple contacts of M3 with pre-M1 and the M4 linkers exist^46,49^. For example, H-bonding of Tyr519 (in M1) to Asp515 (in pre-M1) in response to JNJ-059 could signal through Pro516, which is in van der Waals distance of Phe619 in the M3 gate (and is adjacent to Asp515 in pre-M1). This outlines one potential signaling route leading from the NAM pocket to the gate. We also propose a role for annular lipids in both drug and TARP action—they are consistently observed lining the side of the NAM pocket in AMPAR cryo-EM structures^10^, and mutation of lipid-interacting residues affects NAM activity in this study. Lipid rearrangement in response to NAMs may not only shape the dynamics of pre-M1 but also bridge the NAM pocket with the helices of the conduction path (Supplementary Fig. 5g).

The extracellular TARP loops have been intensively studied in attempts to understand TARP modulation^50,51^, yet our results now highlight the (currently understudied) role of the TARP transmembrane sector^52^. Mutations throughout M1 and M4 result in either positive or negative TARP action, implying that individual γ8 contact points are sensitive to modulation, and can be communicated differently to various points along the conduction path. The upper portion of M1 (encompassing the NAM binding site) faces pre-M1 and the M3 gate and is more loosely connected to γ8^38^, while the more tightly connected lower half of M1 is level with the selectivity filter (Fig. 5d). Interestingly, alanine mutations in the more tightly coupled lower segment generally convey positive TARP action on desensitization while those at the upper M1 end are neutral. Resensitization is affected differently by these mutations, highlighting that these functional components (desensitization versus resensitization) engage different allosteric pathways. These TARP contacts are altered globally as a result of ligand binding to its pocket.

We propose that existing γ8 NAMs could be exploited further through targeted drug design in future efforts. Exposure of the NAM pocket to lipid^53^, together with unoccupied cavities, provide an opportunity for continued development of modulators. For example, in the LY-481 structure, the pocket extends from the oxindole carbonyl toward Ser128 at the kink of the γ8 M2 helix. This region is highly electropositive and accessible to water, with putative water molecules coordinated by the Ser128 side chain (Supplementary Figs. 4c and 5g). In fact, the less bulky tryptophan metabolites isatin and 5-OH-indole penetrate deeper into this space (Supplementary Fig. 7a), and thus might lead the way as starting points for drug design.

Novel modulators will not only include NAM action. The unexpected JNJ-059 PAM response with GluA2 appears to engage a different allosteric route(s). Expression of the PAM phenotype is ~5-fold slower than the NAM effect, and is insensitive to mutation at Tyr523 and Met527, while the equivalent positions in GluA1 blunt JNJ-059 NAM action. Together with a strict requirement for four γ8 subunits, and the inability of γ2 to mediate the effect, we hypothesize that the extracellular, sequence-diverse TARP loops are involved. These project towards the LBDs, which, contrary to the TMD sector, exhibit sequence differences between GluA1 and GluA2. Interaction between these loops and the AMPAR LBDs is gating state-dependent^38^, and is known to regulate desensitization kinetics^46,50,51^. Moreover, TARPs at either the A’C’ or B’D’ sites will have a different reach for the LBDs and the gating linkers^19^. The requirement for four TARPs suggests that A’C’ interactions are essential for positive modulation. One fascinating aspect is why specifically JNJ-059 triggers this response. Further derivatives of this compound may lead to improved PAMs, and thus potentially to drugs that selectively boost cognition in the hippocampus^54^.

## Methods

### cDNA constructs

All cDNA constructs were produced using IVA cloning^55^. Constructs used for structural studies in this paper are the same as previously reported, and are all cloned into pRK5 vectors. To express GluA1/A2_γ8 recombinantly, GluA1 (rat cDNA sequence, flip isoform) was fused with a FLAG tag at the N-terminus, and GluA2 (rat cDNA sequence, V1 to S839, flip isoform, R/G-edited, Q/R-edited) was cloned in a tandem configuration with a GGSGSG linker to TARP γ8 (rat cDNA sequence, E2-K419), a human rhinovirus 3 C (HRV 3 C) protease cleavage site and an eGFP. We note that the AMPAR-TARP fusion constructs, connecting the end of the GluAx M4 helix and the beginning of the TARP M1 helix, has a 40-residue-long unstructured segment, and is therefore not expected to constrain TARP binding and/or action.For electrophysiological recordings tandem constructs containing full-length GluA1/2 and TARP γ8 sequences were used.

### Expression and purification of GluA1/2_γ8

To achieve heteromeric AMPAR expression, FLAG-tagged GluA1 and GluA2-TARPγ8-eGFP tandem plasmids were co-transfected into HEK-Expi293^TM^ cells at a ratio of 1:1. To prevent AMPA-mediated excitotoxicity, AMPAR antagonists ZK200775 (2 nM, Tocris, Cat# 2345) and kynurenic acid (0.1 mM, Sigma, Cat# K335-5G) were added to the culture medium. 36-44 hours post-transfection, cells were harvested and lysed for 3 hours in lysis buffer containing: 25 mM Tris pH 8, 150 mM NaCl, 0.6% digitonin (w/v) (Sigma, Cat# 300410-5 G), 5 μM NBQX, 1 mM PMSF, 1× Protease Inhibitor (Roche, Cat# 05056489001). Insoluble material was then removed by ultracentrifugation (131,000 × *g*, 1 h, rotor 45-50 Ti) and the clarified lysate incubated with anti-GFP beads for 3 h. After washing with glyco-diosgenin (GDN) (Anatrace, Cat# GDN101) buffer (25 mM Tris pH 8, 150 mM NaCl, 0.02% GDN) the protein was eluted from the beads by digestion with 0.01 mg/ml 3 C protease at 4 °C overnight. Eluted fractions were incubated with ANTI-FLAG M2 affinity gel (Sigma, Cat# A2220) for 1.5 h and washed 3 times with GDN buffer. Finally, the complex was eluted using 0.15 mg/ml 3×FLAG peptide (Millipore Cat# F4799) in GDN buffer. Eluted fractions were pooled and concentrated to ~2.5 mg/ml for cryo-EM grid preparation.

### Cryo-EM grid preparation and data collection

Cryo-EM grids were prepared using a FEI Vitrobot Mark IV. For the resting state GluA1/A2_γ8 heteromeric complex, protein was incubated with 300 μM ZK200775 and 40 μM ligands (JNJ-61432059, MedChemExpress, Cat# HY-111751, JNJ-55511118, Tocris, Cat#6278, LY-3130481, MedChemExpress, Cat# HY-108707) for at least 30 min on ice before freezing. For the active state A1/A2_γ8/C2 heteromeric complex, protein was first incubated with 300 μM cyclothiazide (CTZ, Tocris, Cat# 0713) and 40 μM JNJ-059 for at least 30 min on ice and then quickly mixed with 1 M L-glutamate stock solution to a final concentration of 100 mM prior to loading onto the grids. Quantifoil Au 1.2/1.3 grids (300 mesh) were glow-discharged for 30 s at 0.35 mA before use. 3 μl sample was applied to the grids, blotted for 4.5–5 s at 4 °C with 100% humidity and plunge-frozen in liquid ethane. All cryo-EM data were collected using EPU 2.10 on a FEI Titan Krios operated at 300 kV, equipped with a K3 detector (Gatan) and a GIF Quantum energy filter (slit width 20 eV). Movies at 1.5–2.5 μm underfocus were taken in counting mode with a pixel size of 1.07 Å/pixel. A combined total dose of 50 e/Å^2^ was applied with each exposure and 50 frames were recorded for each movie.

### Cryo-EM data processing and model building

Dose-fractionated image stacks were first motion-corrected using MotionCor2^56^. Corrected sums were used for CTF estimation by GCTF^57^. All further data processing was performed with RELION 3.1^58^. Automatic particle picking was performed using a Gaussian blob and particles were binned to 4.28 Å/pixel and extracted in a box of 80 pixels. 2 to 3 rounds of 2D classification were carried out to remove particles not showing AMPAR-like features. For the following 3D classification, emd-4575 was used as initial model to further eliminate low-quality particles. Following data clean-up, particles were re-centered, scaled up to 2.14 Å/pixel and re-extracted in a box of 160 pixels. Another 3D classification was applied on the re-extracted data set. After this round of classification, selected particles were scaled to the original 1.07 Å/pixel size, and refined with C1-symmetry followed by post-processing. CTF refinement and Bayesian polishing were then performed, followed by another refinement. To further improve map resolution, we applied masked refinement on LBD-TMD region and TMD region alone with C2-symmetry introduced. Local resolution was estimated by RELION 3.1.

Model building and refinement were performed using Coot^59^, REFMAC5^60^ and PHENIX^61^ real-space refinement. The GluA1/A2-γ8 complex (PDB 6QKC) was used as starting points, and rigid body-fitted into the maps using UCSF chimera (http://www.rbvi.ucsf.edu/chimera) then automatically refined by REFMAC5. Afterwards, manual refinement was performed through Coot, followed by PHENIX real-space refinement to further refine the geometry. Ligand models were first built manually in Coot by using ligand builder and then fitted into maps. Corresponding restraint files were generated by using PHENIX elbow. After merging ligand and refined protein coordinates, final refinements were carried out by using PHENIX real-space refinement and model validation was performed with MolProbity^62^. LY-481 was built as (-) enantiomer based on a previous study^35^. Graphics were prepared using UCSF Chimera, ChimeraX or PyMOL (http://www.pymol.org). Pore radius was calculated using a plugin version of HOLE^63^ in Coot.

### All-atom MD simulations

Four ligand-bound, and two apo structures of the LBD-TMD-TARP complex described in this and previous studies from the lab^22,38^, were used to set up simulations. Missing residues and loops were added using MODELLER 10.1^64^. The TARP β1-loop is modelled in an extended conformation that avoids intertwining during modelling, by applying distance restraints between Cα atoms of residues 409 and 410 on GluA2 and residues 61 and 62 in the TARP β1-loop, set to be less than 10 Å apart. 100 models were generated, and three models with the highest consensus DOPE score^65^ and SOAP-LOOP score^66^ were used as input structures to prepare the systems for MD simulations. CHARMM-GUI v1.7^67^ was used for system setup. TARP residues N53 and N56 were glycosylated, and TARP E216 was protonated as it is surrounded by hydrophobic residues. CGENFF parameters^68^ were used for all ligands. The protein complexes were embedded in POPC lipid bilayers. TIP3P water was used to solvate the system and 150 mM NaCl was added.

Three simulations were performed for each of the six systems. After minimization for 10,000 steps, two equilibration steps in the NVT ensemble of 125 ps each, an equilibration step in the NPT ensemble of 125 ps with a 1 fs time step, followed by three equilibration steps in the NPT ensemble, each 500 ps with a 2 fs time step, were performed, where harmonic restraints on the protein, and planar/dihedral constraints on the lipids were consecutively decreased. Following removal of all restraints, the NPT ensemble was used for production runs. All unrestrained production runs completed 500 ns, except one of the repeats for JNJ-118 that we had to end at 250 ns due to compute resource constraints; we thus obtained a cumulative of 1.25 microseconds of sampling for JNJ-118, and 1.5 microsecond sampling for all other systems. These simulations were performed using NAMD 3.0 alpha^69^, where the simulation temperature was controlled at 303.15 K by Langevin dynamics, with a damping coefficient of 1 ps^−1^, and the pressure of the system was kept at 101.325 kPa (1.01325 bar or 1 atmosphere) using the Nosé-Hoover Langevin method^70^ with a piston period of 200 fs and piston oscillation decay time of 100 fs. The CHARMM36m force-field^71^ and a 2.0 fs time step for production runs was used for all systems. Analysis of the simulation data and preparation of graphics was done using VMD 1.9.4 a51^72^. All analyses were performed with a sampling of 100 ps/frame. Contact maps were generated by calculating the percentage of simulation time unique inter-residue contacts existed, determined as residues having at least one non-H atom each within a distance of 4 Å of each other, using custom scripts with VMD 1.9.4 a51. The first 100 ns of each simulation was excluded for contacts calculations to allow an extended equilibration time, and resulting percentages were averaged over identical subunits and all three repeat runs. To generate difference maps, the difference in contact percentages between apo and ligand- bound states in the same state, i.e., open vs open and resting vs. resting were taken, excluding any contact percentage <10% as well as difference <10%, to reduce noise.

A single set each of 350 ns long simulations was also performed for only the transmembrane domains of the A1/A2/g8 complex with JNJ-059 and isatin at the ligand site. Isatin was modeled in based on the JNJ-059 oxindole position. The simulation protocol followed was the same as described above for ligand-bound complexes.

### Patch-clamp recording

Glutamate-evoked currents were recorded from transfected HEK293T cells (ATCC, CRL-3216) in the whole-cell patch-clamp configuration at room temperature. The cells were bathed in the extracellular solution consisting of (in mM): NaCl (145), KCl (3), CaCl_2_ (2), MgCl_2_ (1), glucose (10), and HEPES (10), adjusted to pH 7.4 using NaOH. For heteromeric recordings 20 μM IEM 1925 dihydrobromide (Tocris, Cat#4198) was added to the extracellular solution to limit the contribution of GluA1 homomers. Recording pipettes were pulled from thick-walled borosilicate glass capillary tubes (1.5 mm OD, 0.86 mm ID; Science Products GmbH) on a Flaming/Brown puller P-1000 (Sutter Instruments), fire-polished to give a final resistance of 3–6 MΩ and filled with the intracellular solution containing (in mM): CsCl (130), EGTA (10), ATP-sodium salt (2), HEPES (10), and spermine (0.1), adjusted to pH 7.3 with CsOH for whole-cell recordings (junction potential +5.3 mV. Recordings were performed by applying L-glutamate (10 mM) onto lifted cells held at −60 mV transmembrane potential (not corrected for the junction potential). Access resistance in whole-cell recordings was monitored but not compensated. Cells with access resistance unstable or higher than 20 MΩ were excluded from the analysis. Agonist was dissolved in extracellular solution and applied using a double-barrel pipette (#BT-150-10; Science Products GmbH; pulled to a tip diameter of 150–200 μm) mounted on a piezo actuator (Physik Instrumente); each barrel was fed by multiple lines of tubing, via a manifold, to enable recording in the presence or absence of various ligands. Lifted cells were placed ~50 μm from the tip. Data were acquired and filtered (at 5 kHz 4-pole Bessel filter) with MultiClamp 700B amplifier (Molecular Devices), digitized with Digidata 1440 A (Molecular Devices) at a sampling rate of 20 kHz and saved into PC using pClamp10 software (Molecular Devices).

In the whole-cell recordings glutamate was applied to the cells in 2 s pulses every 5 s. Control currents were recorded for at least 60 s then tested compounds were applied during and between glutamate pulses for 120 s and then washed off. Coverslips with the cells were removed after each application of compound to avoid recording from cells pre-exposed to compound. The time-course of macroscopic desensitization was measured by fitting the decay phase of the currents from 95% to the steady-state current with one or two exponentials; when two exponentials were used, weighted time constant of desensitization was calculated as follows: τ_w,des_ = τ_f_ (A_f_/(A_f_ + A_s_)) + τ_s_ (A_s_/(A_f_ + A_s_)), where τ_f(s)_ and A_f(s)_ represent the fast(slow) component time constant and coefficient, respectively. Equilibrium current was measured as steady-state current percentage of peak current. The resensitization was defined as an excess steady-state current following desensitization through to the end of 2 s glutamate pulse and expressed as the percentage of peak current. Changes in peak current were quantified by measuring and averaging (5 sweeps) peak amplitude just before the application of the ligand and at the full extent of the modulatory effect: at 1 min or 2 min after ligand application for negative or positive modulation, respectively. Modulatory compounds were dissolved in DMSO to 50 mM as stock solution and used at final concentration of 10 µM.

### Quantification and statistical analysis

Summary data are presented as mean ± standard error of the mean (SEM). Statistical tests were performed using GraphPad Prism 9.4.0. *p* values were calculated from one- or two-sample (paired or unpaired) two-tailed Student’s *t*-tests. Ordinary one-way ANOVA test with Dunnett correction was used for multiple comparisons. *p* values in the figures are indicated as *p ≤ 0.05, **p ≤ 0.01, ***p ≤ 0.001 and ‘ns’ for *p* > 0.05; exact *p* values and further details of the statistical tests are provided in the Source Data.

### Reporting summary

Further information on research design is available in the Nature Portfolio Reporting Summary linked to this article.

## Supplementary information

Supplementary Information

Description of Additional Supplementary Files

Supplementary Movie 1

Supplementary Movie 2

Reporting Summary

## Data Availability

The data that support this study are available from the corresponding authors upon request. The cryo-EM maps have been deposited in the Electron Microscopy Data Bank (EMDB) under accession codes EMDB-15714 (A1/2 γ8 + JNJ-059 resting state), EMDB-15716 (A1/2 γ8 + JNJ-118 resting state), EMDB15717 (A1/2 γ8 + LY-481 resting state), and EMDB-15718 (A1/2 γ8 + JNJ-059 open state). The coordinates have been deposited in the Protein Data Bank (PDB) under accession code 8AYL (A1/2 γ8 + JNJ-059 resting state), 8AYM (A1/2 γ8 + JNJ-118 resting state), 8AYN (A1/2 γ8 + LY-481 resting state), and 8AYO (A1/2 γ8 + JNJ-059 open state). Previously published structures are available from RCSB Protein Data Bank with accession codes 7QHB, 7OCD, 7OCE, and 6QKC. The source data underlying Figs. 4b, c, 5c, 6a–e, and Supplementary Figs. 6a–b, 7a, 8a–c, and 9a–c are provided as a Source Data file. Source data are provided with this paper.

## Source data

Source Data
