# Supplementary material for: Modulatory mechanisms of TARP γ8-selective AMPA receptor therapeutics
Source: Nat Commun. 2023 Mar 25;14:1659. doi: 10.1038/s41467-023-37259-5 (PMC10039940; doi:10.1038/s41467-023-37259-5)

Supplementary Information

Modulatory mechanisms of TARP γ8-selective AMPA receptor therapeutics

Danyang Zhang, Remigijus Lape, Saher A. Shaikh, Bianka K. Kohegyi, Jake F. Watson, Ondrej Cais, Terunaga Nakagawa, Ingo H. Greger

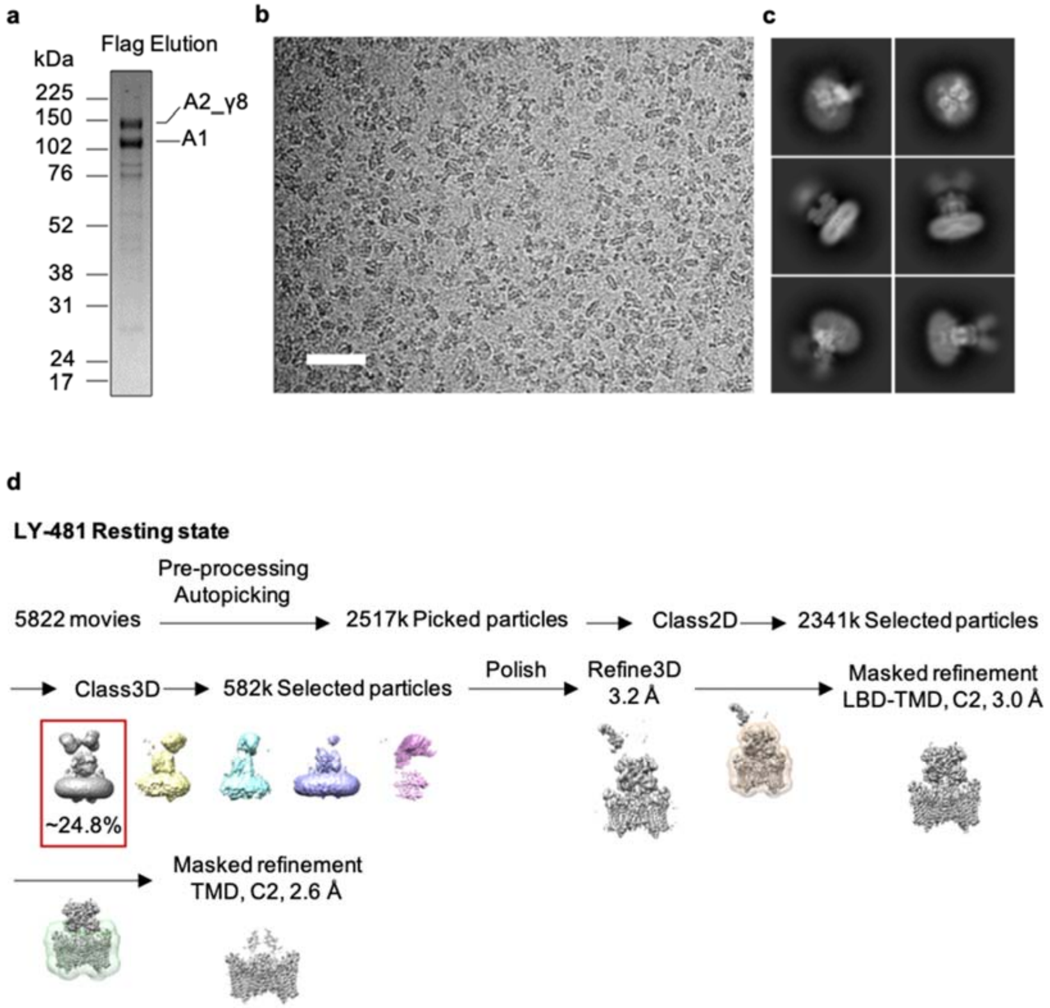

Supplementary Fig. 1. Purification of recombinant AMPAR complex and representative cryo-EM data processing workflow of GluA1/A2_γ8 in complex with γ8 NAMs.

a, Representative 4-12% Bis-Tris SDS-PAGE gel stained with Coomassie blue, indicating elution of the GluA1/2_γ8 complex from FLAG beads. Purification was performed reproducibly (more than 3 times); uncropped gel provided at the end of Supplementary Information. **b**, Representative, motion-corrected micrograph of resting-state GluA1/2_γ8 in complex with LY-481 among collected data (scale bar, 50 nm). **c**, Representative 2D class averages of the resting state GluA1/2_γ8 in complex with LY-481. **d**, Cryo-EM data processing workflow of the resting state GluA1/2_γ8 in complex with LY-481. Raw movies were first

processed, then more than 2 million raw particles were picked from motion-corrected micrographs. Then 2D and 3D classification was performed to remove bad particles, and finally 582k particles were selected and polished for refinement. Next, focused refinement on LBD-TMD gating core was performed with C2 symmetry. To further improve the resolution at the ligand-binding pocket, the TMD sector alone was refined with C2 symmetry applied. This workflow was also implemented for the other structures (JNJ-118, JNJ-059).

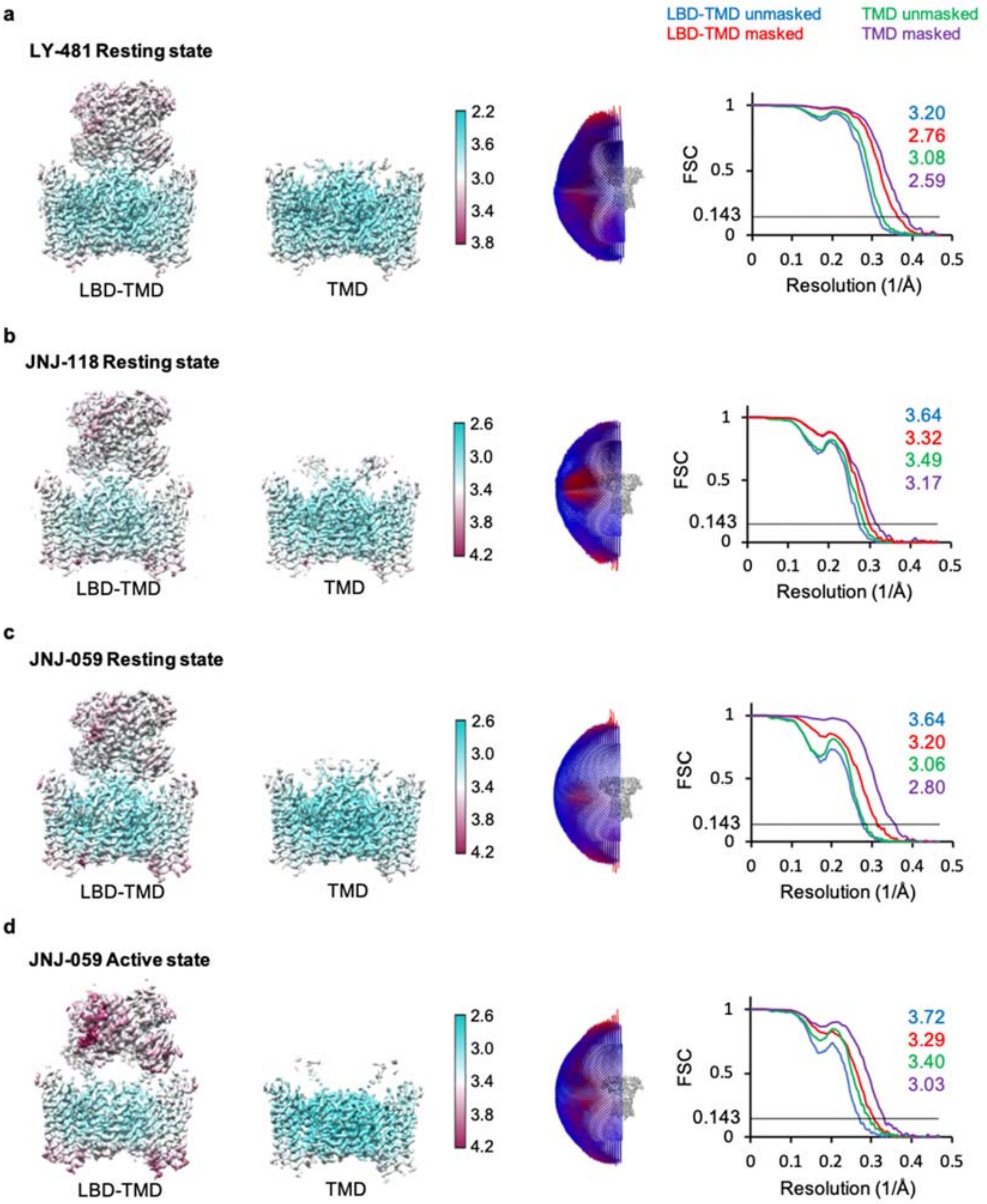

Supplementary Fig. 2. Cryo-EM analysis of GluA1/A2_γ8 in complex with γ8 NAMs.

a, **Left:** Local resolution maps at LBD-TMD and TMD of resting state GluA1/A2_γ8 in complex with LY481. 3D maps are coloured based on local resolution estimate. **Middle:** Euler angle distribution of particles used for the cryo-EM reconstruction. **Right:** Masked (red, LBD-

TMD and purple, TMD) or unmasked (blue, LBD-TMD and green, TMD) Fourier shell correlation (FSC) of corresponding maps where FSC=0.143 (black line). **b**, Local resolution maps, particle Euler angle distribution and FSC curves of resting state GluA1/A2_γ8 in complex with JNJ-118. Figures are coloured as in A. **c**, Local resolution maps, particle Euler angle distribution and FSC curves of resting state GluA1/A2_γ8 in complex with JNJ-059. Figures are coloured as in A. **d**, Local resolution maps, particle Euler angle distribution and FSC curves of open state GluA1/A2_γ8 in complex with JNJ-059. Figures are coloured as in panel A.

49

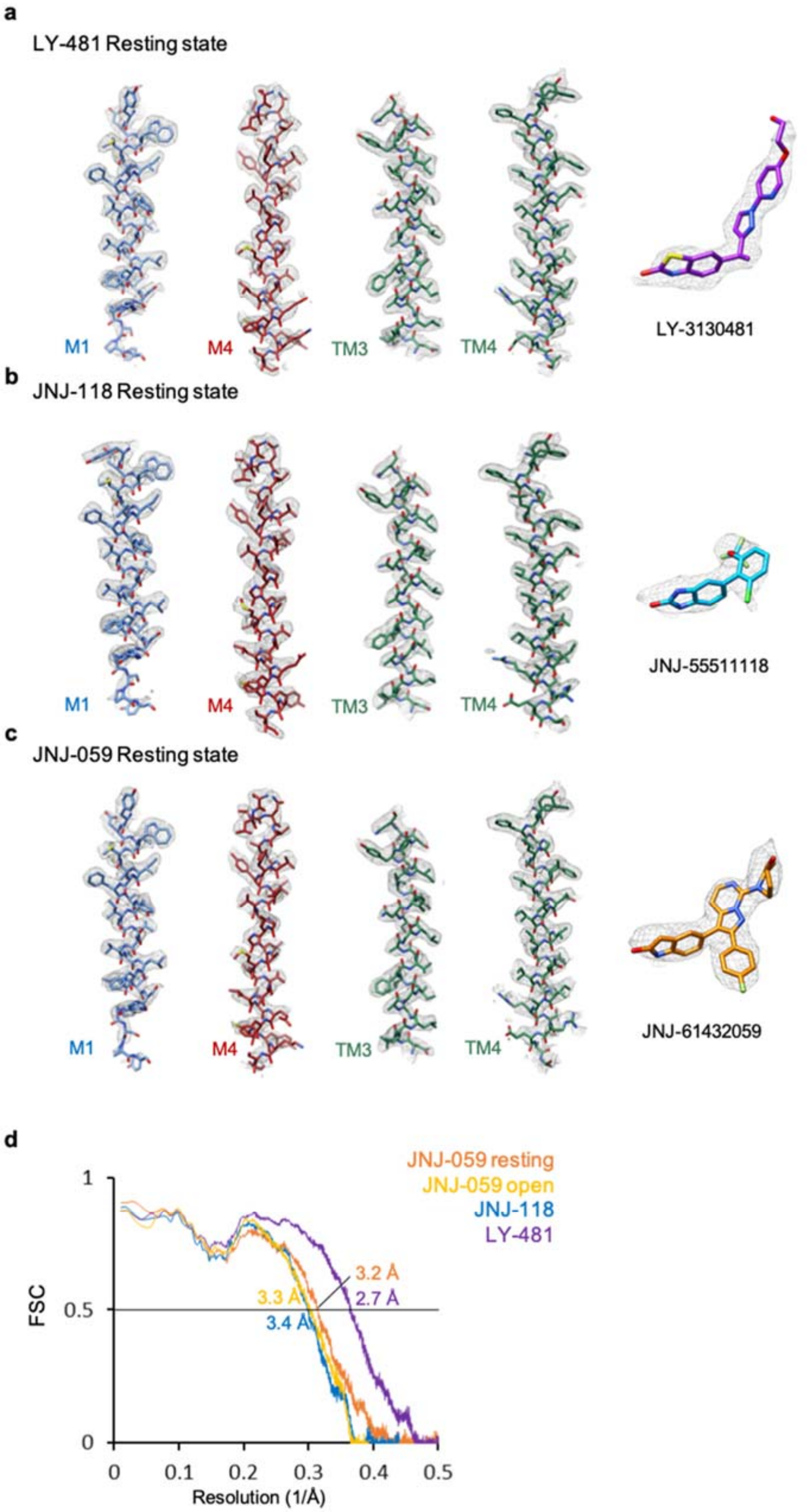

a
LY-481 Resting state
M1
M4
TM3
TM4
LY-3130481
b
JNJ-118 Resting state
M1
M4
TM3
TM4
JNJ-55511118
c
JNJ-059 Resting state
M1
M4
TM3
TM4
JNJ-61432059
d
JNJ-059 resting
JNJ-059 open
JNJ-118
LY-481
FSC
1
0.5
0
3.2 Å
3.3 Å
2.7 Å
3.4 Å
0
0.1
0.2
0.3
0.4
0.5
Resolution (1/Å)

50

Supplementary Fig. 3. Densities and their fit against models of GluA1/A2_γ8 transmembrane helices.

a, Densities of LY-481 and surrounding transmembrane helices M1(GluA1), M4(GluA2), M3(γ8), M4(γ8) and their fit against the model. **b**, Densities of JNJ-118 and surrounding transmembrane helices M1(GluA1), M4(GluA2), M3(γ8), M4(γ8) and their fit against the model. **c**, Densities of JNJ-059 and surrounding transmembrane helices M1(GluA1), M4(GluA2), M3(γ8), M4(γ8) and their fit against the model. **d**, Model-to-map FSCs of ligand-bound GluA1/2_γ8 LBD-TMD models in resting and active states.

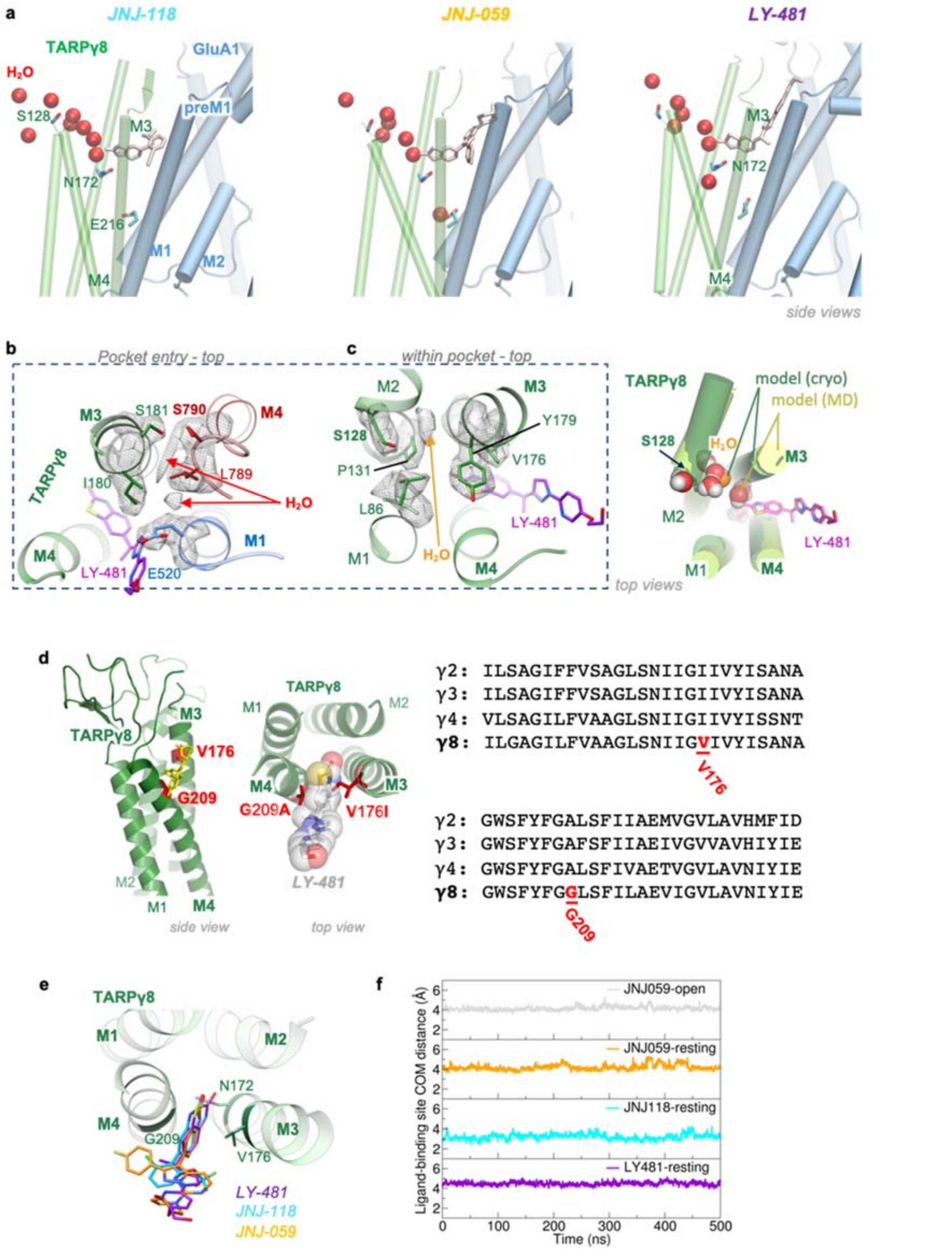

Supplementary Fig. 4. Features of the NAM binding sites from cryo-EM and MD simulation analysis.

a, Snapshots from MD simulations (left: JNJ-118, centre, JNJ-059, right: LY-481) showing water molecules from the extracellular side penetrating into TARP in the direction of S128 to N172 at the ligand binding site. **b**, Density of putative waters (indicated by the red arrow) and surrounding residues in the LY-481 cryo-EM map around GluA2 S790. Model is coloured as in Fig2. **c**, Left: Density of putative waters (indicated by the red arrows) and surrounding residues in the LY-481 cryo-EM map around γ8 S128. Model is coloured as in Fig2. Right: Top view of MD simulation snapshot showing water penetration into TARP in the LY-481 complex, in the S128 to N172 region. **d**, Sequence alignment of Type 1 TARPs, γ8-specific V176 and G209 are highlighted. The model demonstrates how these residues accommodate the ligands, in contrast with the bulkier residues of the other TARPs, shown in the top view in red. **e**, Overlay of resting state LY-481, JNJ-118 and JNJ-059 models. Models are coloured as in Fig2. **f**, Ligand stability in the binding site, measured as the centre of mass (COM) distance of ligand heavy atoms from COM of Cα of binding site residues, V176, G209, M523 and C524. Representative variations from one binding site in one simulation set for each system are shown. Low variation indicates the ligands remain bound in the site during simulations.

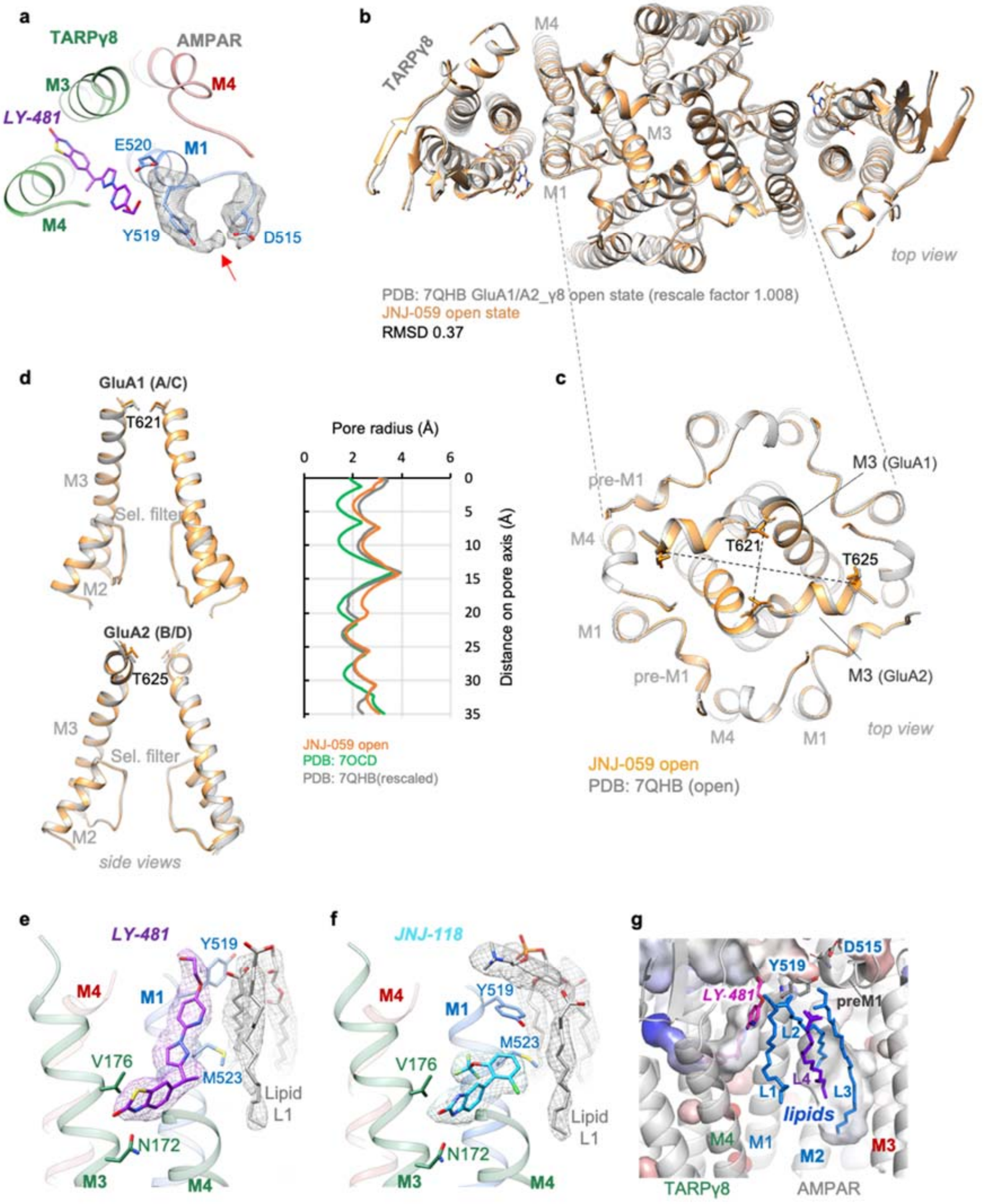

Supplementary Fig. 5. JNJ-059 open state and annular lipids.

a, Density of putative water that bridges Y519 and D515 in the LY-481 cryo-EM map. Model is coloured as in Fig2. **b**, Superposition of open state GluA1/A2_γ8 in complex with JNJ-059 (orange) and the open state GluA1/A2_γ8 without ligand binding (PDB 7QHB; the model was

rescaled by a factor of 1.008 to eliminate systematic errors caused by pixel size calibration yielding better comparability). **c**, Zoomed-in view of the superposed models shows subtle changes at the receptor gate with JNJ-059 bound. **d**, Pore dimensions of resting state GluA1/A2_γ8 (green, PDB 7OCD) open state GluA1/A2_γ8 (grey, rescaled 7QHB as in panel B), and open state GluA1/A2_γ8 in complex with JNJ-059 (orange) depicted by space-filling representation. Side views of superposed M3 helices (top: GluA1, bottom: GluA2) from open state models are shown. **e**, Density of LY-481 and the lipid molecules surround ligand-binding pocket. Model is coloured as in Figure2. **f**, Density of JNJ-118 and lipid molecules surround the ligand binding pocket. Model is coloured as in Fig2. **g**, Cavities of the NAM (LY-481 serves as example) and annular lipids lining the conduction path helices (M2 and M3).

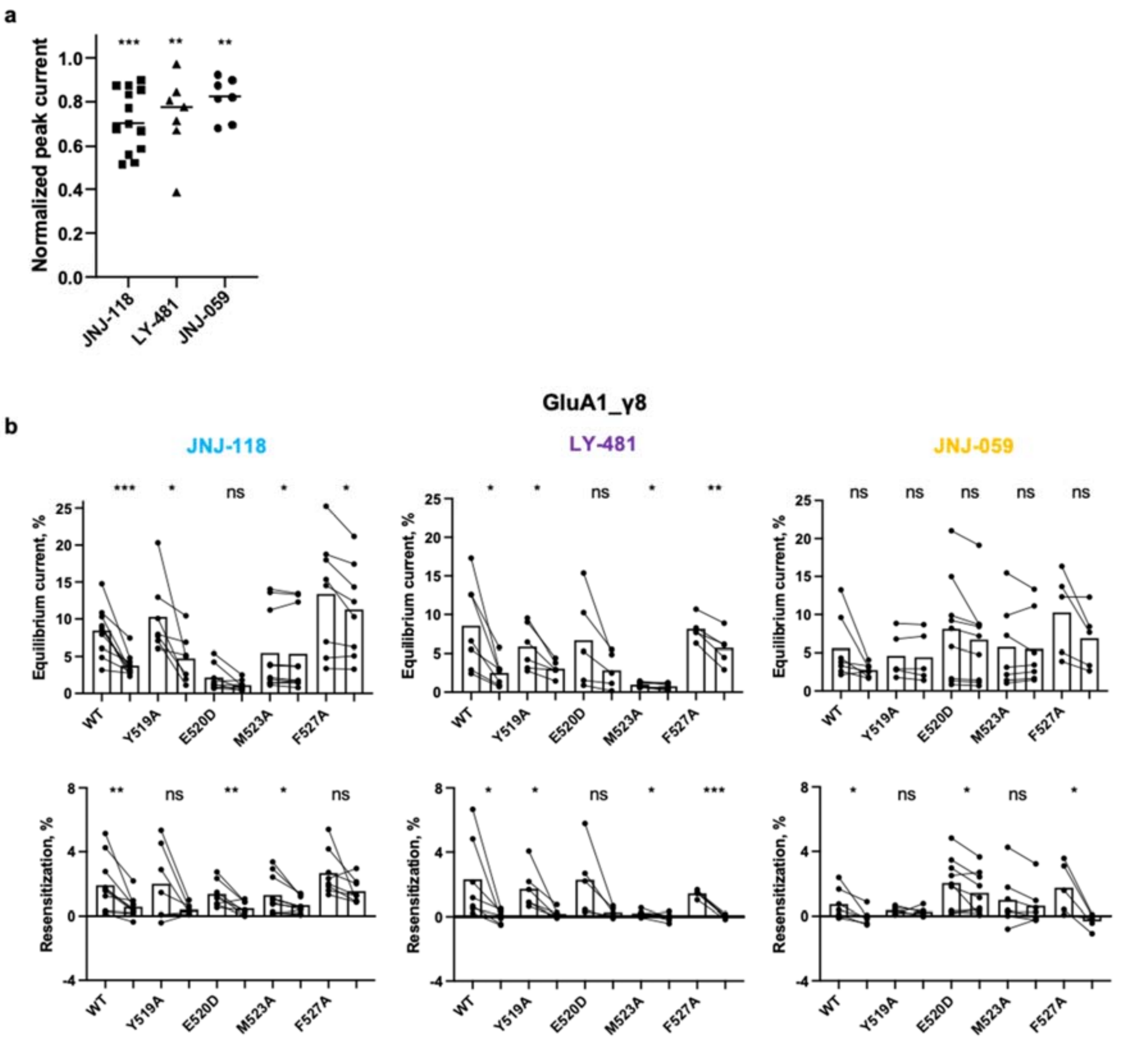

Supplementary Fig. 6. Electrophysiological characterisation of GluA1 wt and mutants in response to the three NAMs.

a, Pooled scatter plot of GluA1i_γ8 current peak inhibition by three modulators. Each point is current peak in the presence of modulator normalized to the control peak. Horizontal lines indicate the mean values. Asterisks summarize two-tailed one-sample t-test (difference from 100%) results: ** $p <= 0.01$, *** $p <= 0.001$. Number of cells: n=13, 7, and 7 for JNJ-118, LY-481, and JNJ-059, respectively. **b**, Paired bar plots showing effect of 10 µM JNJ-118, LY-481 or JNJ-059 on equilibrium current and resensitization for wild-type or mutant GluA1i_γ8. Each point is a measure of parameter in absence or presence of modulator. Bar height represents the

mean value. Asterisks indicate summary of two-tailed paired t-test values: * $p \leq 0.05$, ** $p \leq 0.01$, *** $p \leq 0.001$ and ‘ns’ for $p>0.05$. Number of cells: JNJ-118: n=10, 7, 8, 10, and 8; LY-481: n=7, 6, 5, 8, and 5; JNJ-059: n=7, 5, 9, 7, and 5 for wt, Y519A, E520D, M523A, and F527A, respectively. Source data are provided as a Source Data file.

114

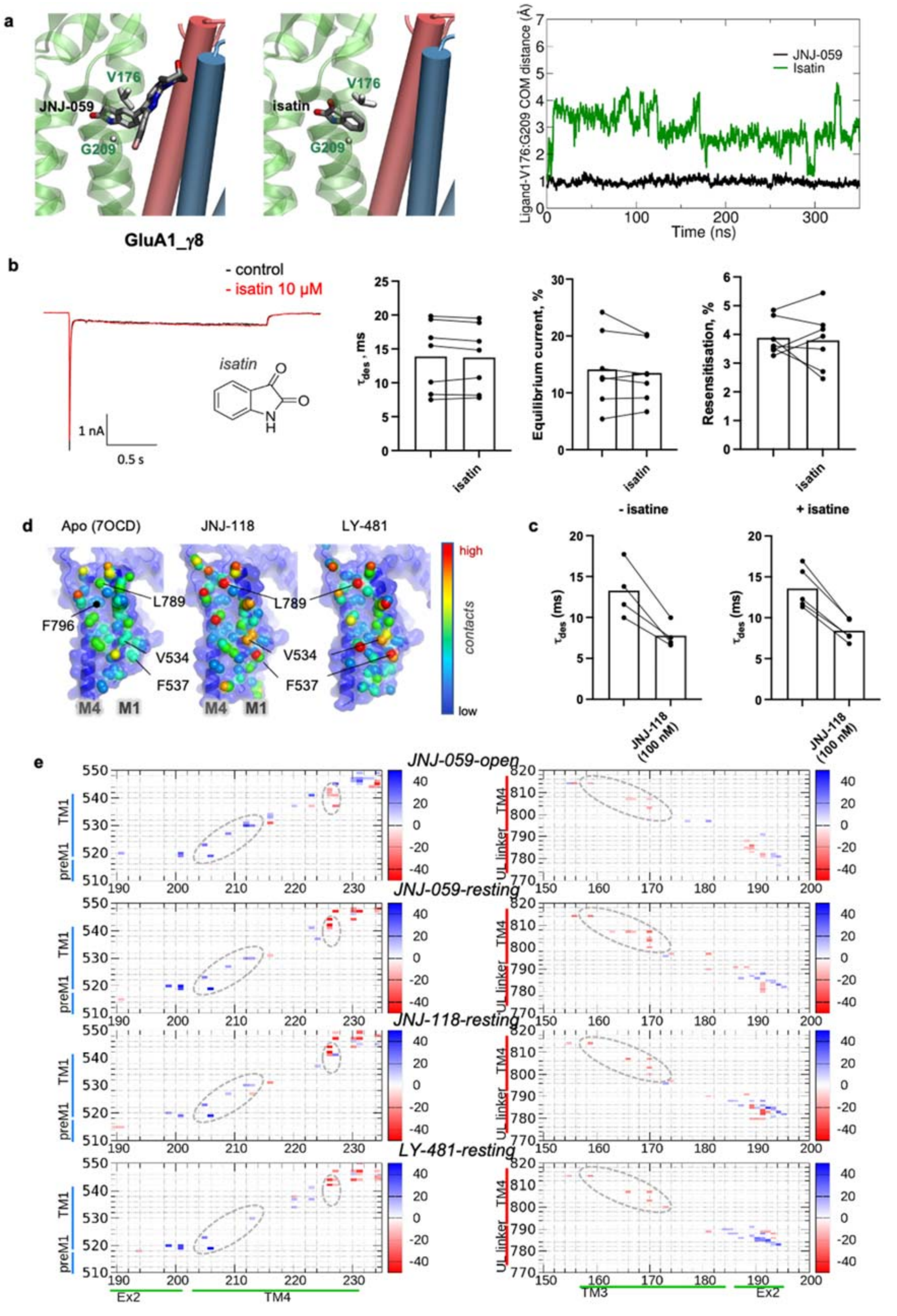

a
JNJ-059
V176
G209
isatin
GluA1_γ8
Ligand-V176:G209 COM distance (Å)
Time (ns)
JNJ-059
Isatin
b
- control
- isatin 10 μM
isatin
1 nA
0.5 s
τdes , ms
Equilibrium current, %
Resensitisation, %
d
Apo (7OCD)
JNJ-118
LY-481
L789
F796
V534
F537
M4
M1
high
contacts
low
c
- isatine
+ isatine
τdes (ms)
JNJ-118 (100 nM)
e
JNJ-059-open
JNJ-059-resting
JNJ-118-resting
LY-481-resting
TM1
preM1
TM4
UL_linker
Ex2
TM3

115

Supplementary Fig. 7. Role of natural oxindoles and contact map analyses.

a, MD snapshots after initial equilibration runs (before production runs) showing the positioning of JNJ-059 (left) and isatin (centre) in the TARP binding site, with the oxindole/isatin sandwiched between V176 and G209. Oxindole/isatin stability in the binding site, measured as the centre of mass (COM) distance of oxindole group heavy atoms, from COM of Cα of V176 and G209. **b**, **Left**: Representative whole-cell responses to 10 mM glutamate (2 seconds, −60 mV) from HEK293T cells transfected with GluA1i_γ8 tandem in control condition (black) and in presence of isatin 10 μM (red). **Right**: Paired bar plots showing effect of 10 μM isatin on desensitization τ, equilibrium current and resensitization for GluA1i_γ8. Each point is a measure of parameter in absence or presence of isatin. Bar height represents the mean value (n = 7). Two-tailed paired t-test *p* values for all parameters were *p*>0.05. **c**, Paired bar plots showing effect of 100 nM JNJ-118 on GluA1i_γ8 desensitization τ in absence or presence of 1 mM isatin. Bar height represents the mean value (n = 4 and 5, respectively). **d**, TARP γ8 contact points along its binding site, the $M4_{GluA2}$ and $M1_{GluA1}$ helices. Contacted residues are coloured depending on the number of atoms contributing to the interaction (red: high; blue: low). Contacts were computed using 'findNeighbors' in ProDy' with a 4.5 Å cutoff between heavy atoms (Bakan et al., 2011). **e**, Contact-difference maps (see Methods) for GluA1-TARP (left panel) and GluA2-TARP (right panel) from simulations. Positive values (blue) indicate contacts that are longer lived in apo vs. ligand-bound states, negative values (red) show contacts that are more persistent in ligand-bound systems compared to apo. Dotted ovals highlight the main changes in interfacial contacts from apo to ligand-bound states: ligand binding reduced TARP contact with GluA1 M1 in the top half (blue contacts in oval), but increased contact near the helix base (red contacts in oval). For GluA2

M4, ligand binding induces an overall increase in contact with TARP (red contacts in oval). Source data are provided as a Source Data file.

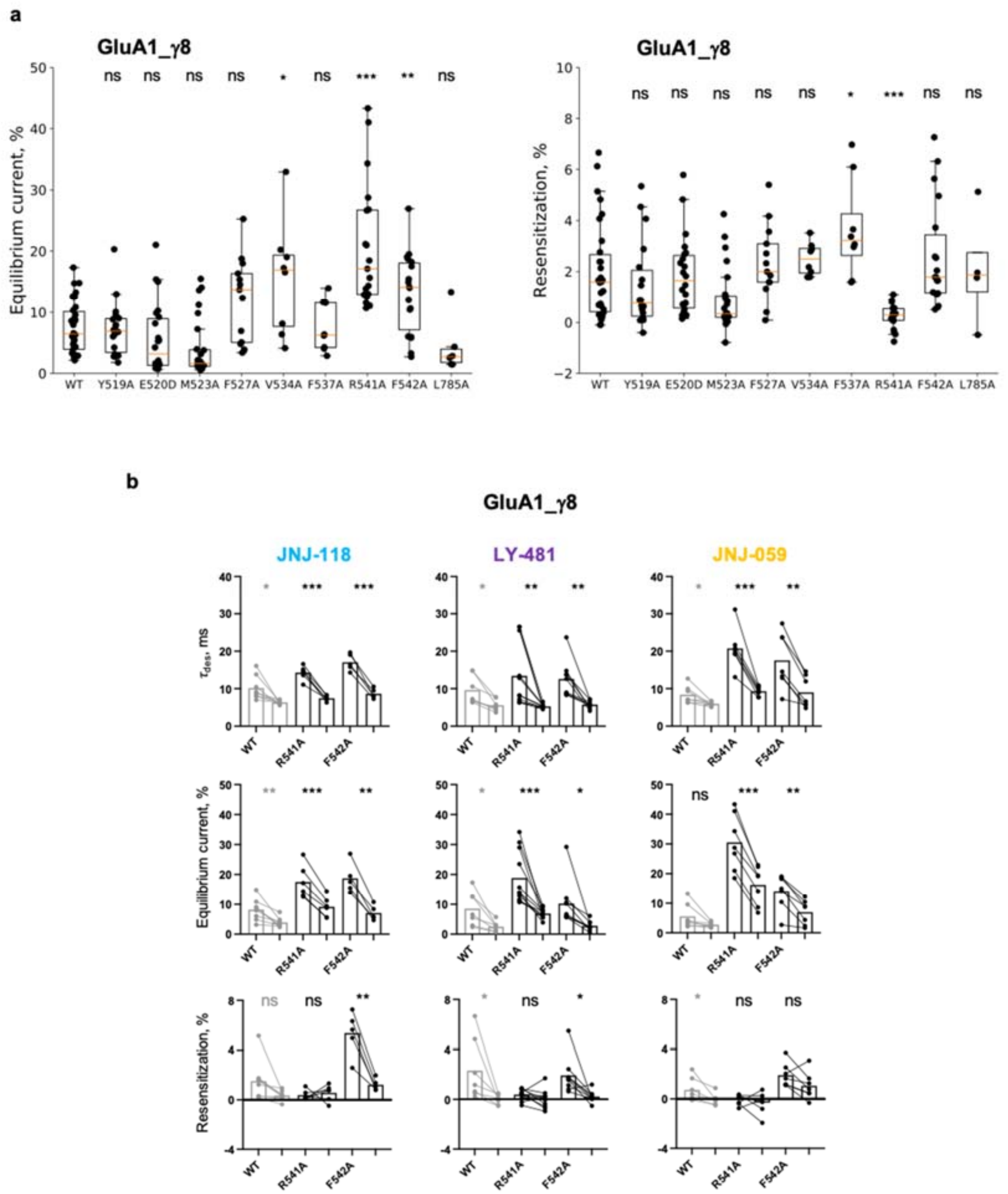

Supplementary Fig. 8. Electrophysiological analysis of GluA1 mutants in response to the three NAMs.

a, Box plots showing equilibrium current and resensitization, for wild type GluA1i_γ8 and several GluA1i mutants as indicated. Boxes show the 25th/75th percentiles and whiskers indicate the furthest points that fall within 1.5 times of interquartile range from the 25th/75th percentiles. The horizontal line in each box shows the median value. Asterisks summarize one-

way ANOVA test with Dunnett correction was used for multiple comparisons to wild type receptor (* $p <= 0.05$, ** $p <= 0.01$, *** $p <= 0.001$ and 'ns' for p>0.05; n values same as in Fig. 5d). **b**, Paired bar plots showing effect of 10 μM JNJ-118, LY-481 or JNJ-059 on desensitization τ, equilibrium current and resensitization for wild type or mutated (R541A or F542A) GluA1i_γ8. Each point is a measure of parameter in absence or presence of modulator. Bar height represents the mean value. Asterisks indicate summary of two-tailed paired t-test values: * $p <= 0.05$, ** $p <= 0.01$, *** $p <= 0.001$ and 'ns' for p>0.05; number of cells: JNJ-118: 8, 6, 5 (for WT, R541A, and F542A, respectively); LY-481: 7, 11, 9; JNJ-059: 7, 7, 7. Greyed out data sets have been presented elsewhere in this paper (Fig. 4b, Fig. S6b). Source data are provided as a Source Data file.

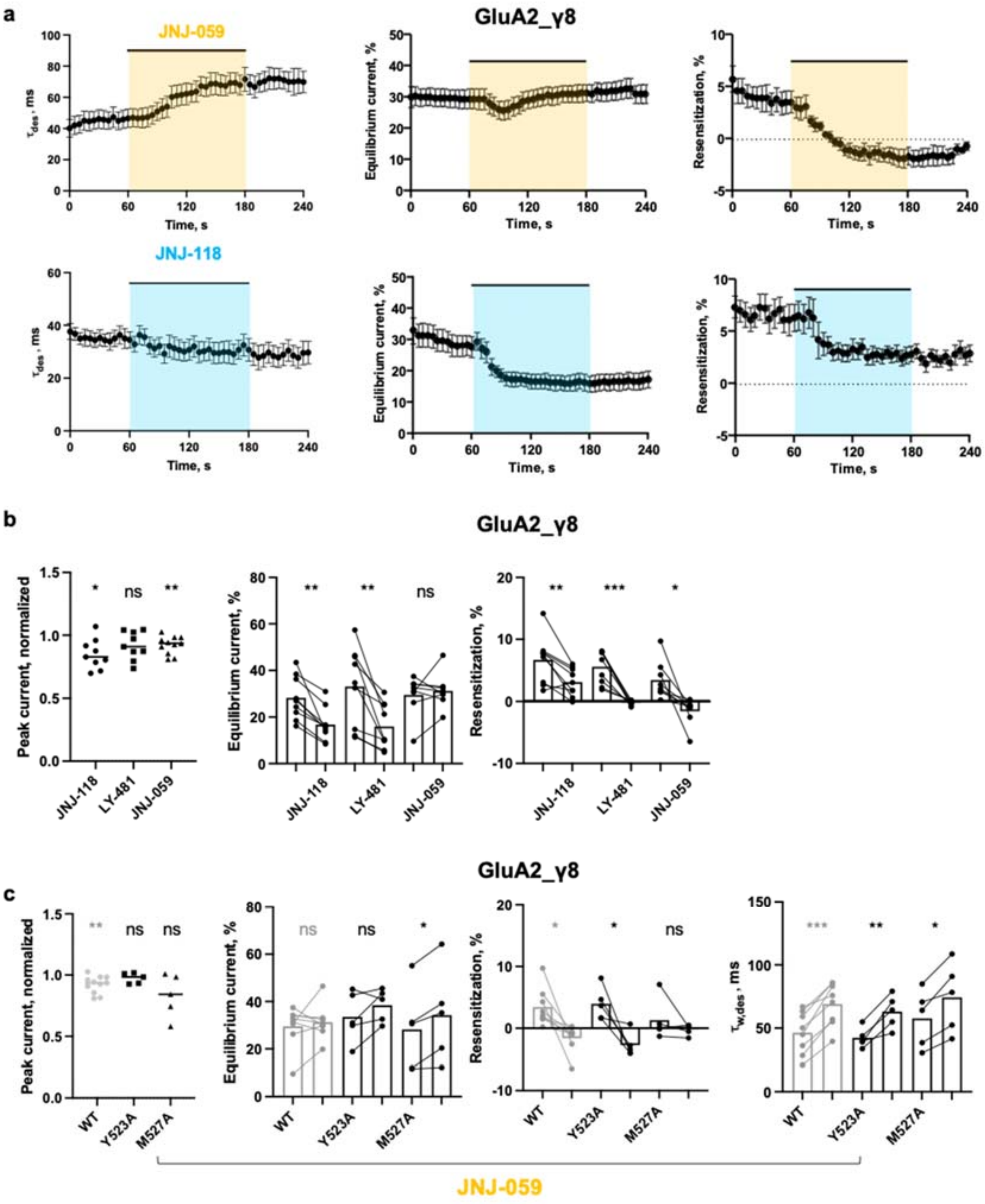

Supplementary Fig. 9. Electrophysiological analysis of GluA2 mutants in response to the three NAMs.

a, Scatter plot of average desensitization τ (left), equilibrium current (middle) and resensitization (right) in time indicating the time-course of JNJ-059 (n=8; top) or JNJ-118 (n=9; bottom) effect on wild-type GluA2iQ_ γ8 receptor currents. Black circles and whiskers indicate

mean values and standard error. **b**, **Left**: Pooled scatter plot of GluA2iQ_γ8 current peak inhibition by three modulators. Each point is current peak in the presence of modulator normalized to the control peak. Horizontal lines indicate the mean values (n = 9, 9, and 11 for JNJ-118, LY-481, and JNJ-059, respectively). Asterisks summarize two-tailed one-sample t-test (difference from 100%) results: * $p <= 0.05$, ** $p <= 0.01$. **Right**: Paired plots showing effect of 10 μM JNJ-118, LY-481 or JNJ-059 on equilibrium current and resensitization for GluA2iQ_γ8. Bar height represents the mean value (n = 9, 9, and 8 for JNJ-118, LY-481, and JNJ-059, respectively). **c**, **Left**: Pooled scatter plot of wild type or mutant GluA2iQ_γ8 current peak inhibition by JNJ-059. Each point is current peak in the presence of JNJ-059 normalized to the control peak. Horizontal lines indicate the mean values (n = 11, 5, and 5 for WT, Y523A, and M527A, respectively). Asterisks summarize two-tailed one-sample t-test (difference from 100%) results: ** $p <= 0.01$ **Right**: Paired plots showing effect of JNJ-059 on equilibrium current, resensitization, and desensitization τ for GluA2iQ_γ8. Bar height represents the mean value (n = 8, 5, and 5 for WT, Y523A, and M527A, respectively). Asterisks indicate summary of one-sample t-test (difference from 1; normalized peak) or two-tailed paired t-test values (paired plots): * $p <= 0.05$, ** $p <= 0.01$, *** $p <= 0.001$ and 'ns' for p>0.05. Greyed out data sets are repeated from **b** and Fig. 6b. Source data are provided as a Source Data file.

Supplementary Table 1.

Cryo-EM data collection, refinement and validation statistics.

	**A1/2_γ8 + LY-481**	**A1/2_γ8 + JNJ-118**	**A1/2_γ8 + JNJ-059**	**A1/2_γ8 + JNJ-059**
	**Resting state**	**Resting state**	**Resting state**	**Open state**
	LBD-TMD<br>(EMDB-15717)<br>(PDB 8AYN)	LBD-TMD<br>(EMDB-15716)<br>(PDB 8AYM)	LBD-TMD<br>(EMDB-15714)<br>(PDB 8AYL)	LBD-TMD<br>(EMDB-15718)<br>(PDB 8AYO)
**Data collection and processing**				
Microscope	FEI Titan Krios	FEI Titan Krios	FEI Titan Krios	FEI Titan Krios
Detector	K3 + GIF	K3 + GIF	K3 + GIF	K3 + GIF
Magnification	81000X	81000X	81000X	81000X
Voltage (kV)	300	300	300	300
Electron exposure (e-/Å2)	50	50	50	50
Defocus range (μm)	-1.2 to -2.8	-1.2 to -2.8	-1.2 to -2.8	-1.2 to -2.8
Pixel size (Å/pixel)	1.07	1.07	1.07	1.07
Symmetry imposed	C2	C2	C2	C2
Micrographs	5822	4019	3781	13612
Map resolution (Å)	2.8	3.3	3.2	3.3
FSC threshold	0.143	0.143	0.143	0.143
**Refinement**				
Initial model used (PDB)	7OCD	7OCD	7OCD	7OCD
Model resolution (Å)	2.8	3.3	3.2	3.3

FSC threshold	0.5	0.5	0.5	0.5
Map sharpening B factor (Å^2)	-90	-104	-88	-106
Model composition				
Non-hydrogen atoms	15748	15962	15702	14512
Protein residues	1998	2008	1998	1962
Ligands	LY-481: 2, ZK: 4	JNJ-118: 2, ZK: 4	JNJ-059: 2, ZK: 4	JNJ-059: 2, CTZ: 4
Lipids	22	22	18	16
B factors (Å2)				
Protein	45.86	34.91	52.15	50.23
Ligand	23.32	17.75	33.71	36.84
R.m.s. deviations				
Bond lengths (Å)	0.008	0.006	0.007	0.007
Bond angles	0.646	0.796	0.608	0.583
Validation				
Molprobity score	1.56	1.01	1.61	1.40
Clashscore	5.41	1.59	5.82	6.56
Poor rotamers (%)	0	0.13	0	0
Ramachandran plot				
Favoured (%)	96.08	97.47	95.83	97.81
Allowed (%)	3.92	2.53	4.17	2.19
Disallowed (%)	0	0	0	0

Uncropped version of the gel presented in Supplementary Fig. 1:

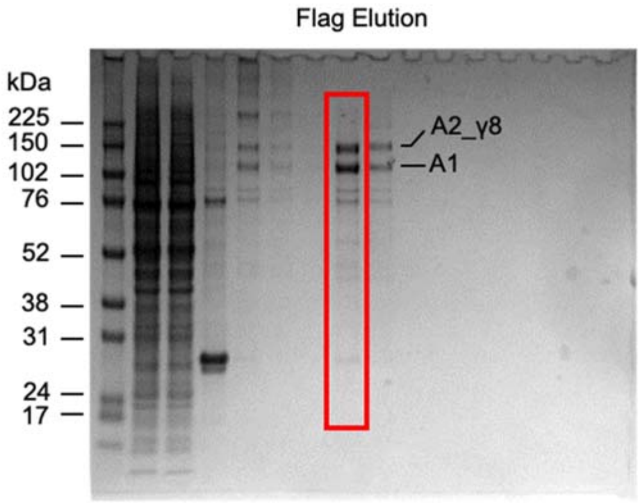

Supplement: Supplementary file 1 — Supplementary Information [file 41467_2023_37259_MOESM1_ESM.pdf]
