# Supplementary material for: Modulatory mechanisms of TARP γ8-selective AMPA receptor therapeutics
Source: Nat Commun. 2023 Mar 25;14:1659. doi: 10.1038/s41467-023-37259-5 (PMC10039940; doi:10.1038/s41467-023-37259-5)
Supplement: Supplementary file 2 — Description of Additional Supplementary Files [file 41467_2023_37259_MOESM2_ESM.pdf]

Description of Additional Supplementary Files

File Name: Supplementary Movie 1
Description: **Cryo-EM map of the LY-481 resting state GluA1/2_γ8 complex, outlining the NAM binding pocket (side and front views).** Colour code: GluA1-blue, GluA2-red, TARP γ8- green, LY-481-purple, lipids-grey, ligand coordinating side chains are shown in stick.

File Name: Supplementary Movie 2
Description: **All-atom MD simulation of the LY-481 resting state GluA1/2_γ8 complex. Colour** code: GluA1-blue, GluA2-red, TARP γ8-green, LY-481-yellow (spheres).
